# HMGA1 induces FGF19 to drive pancreatic carcinogenesis and stroma formation

**DOI:** 10.1172/JCI151601

**Published:** 2023-03-15

**Authors:** Lionel Chia, Bowen Wang, Jung-Hyun Kim, Li Z. Luo, Shuai Shuai, Iliana Herrera, Sophia Y. Chen, Liping Li, Lingling Xian, Tait Huso, Mohammad Heydarian, Karen Reddy, Woo Jung Sung, Shun Ishiyama, Gongbo Guo, Elizabeth Jaffee, Lei Zheng, Leslie M. Cope, Kathy Gabrielson, Laura Wood, Linda Resar

**Affiliations:** 1 Pathobiology Graduate Program, Department of Pathology and; 2 Division of Hematology, Department of Medicine, Johns Hopkins University School of Medicine, Baltimore, Maryland, USA.; 3 Biochemistry and Molecular Biology Program, Johns Hopkins Bloomberg School of Public Health, Baltimore, Maryland, USA.; 4 Department of Surgery,; 5 Department of Biological Chemistry,; 6 Department of Pathology,; 7 Department of Molecular and Comparative Pathobiology,; 8 Department of Oncology, and; 9 Division of Biostatistics, Sidney Kimmel Comprehensive Cancer Center, Johns Hopkins University School of Medicine, Baltimore, Maryland, USA.

**Keywords:** Oncology, Cancer, Growth factors, Oncogenes

## Abstract

High mobility group A1 (HMGA1) chromatin regulators are upregulated in diverse tumors where they portend adverse outcomes, although how they function in cancer remains unclear. Pancreatic ductal adenocarcinomas (PDACs) are highly lethal tumors characterized by dense desmoplastic stroma composed predominantly of cancer-associated fibroblasts and fibrotic tissue. Here, we uncover an epigenetic program whereby HMGA1 upregulates FGF19 during tumor progression and stroma formation. HMGA1 deficiency disrupts oncogenic properties in vitro while impairing tumor inception and progression in KPC mice and subcutaneous or orthotopic models of PDAC. RNA sequencing revealed HMGA1 transcriptional networks governing proliferation and tumor-stroma interactions, including the *FGF19* gene. HMGA1 directly induces *FGF19* expression and increases its protein secretion by recruiting active histone marks (H3K4me3, H3K27Ac). Surprisingly, disrupting FGF19 via gene silencing or the FGFR4 inhibitor BLU9931 recapitulates most phenotypes observed with HMGA1 deficiency, decreasing tumor growth and formation of a desmoplastic stroma in mouse models of PDAC. In human PDAC, overexpression of *HMGA1* and *FGF19* defines a subset of tumors with extremely poor outcomes. Our results reveal what we believe is a new paradigm whereby HMGA1 and FGF19 drive tumor progression and stroma formation, thus illuminating FGF19 as a rational therapeutic target for a molecularly defined PDAC subtype.

## Graphical abstract

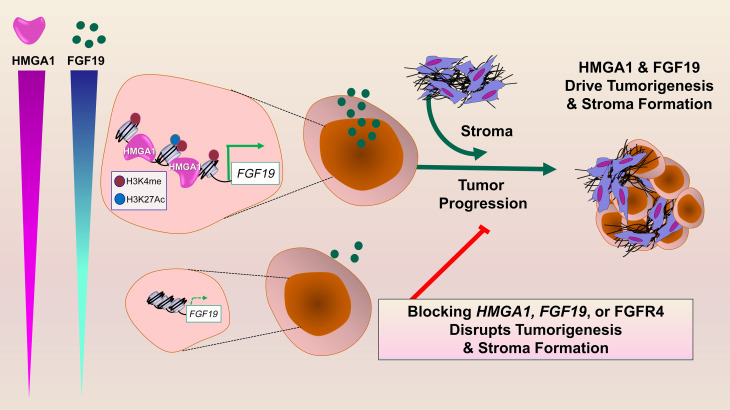

## Introduction

Pancreatic ductal adenocarcinoma (PDAC) has emerged as a major public health problem in industrialized countries, and its incidence is rising (1–3). PDAC is predicted to become the second leading cause of cancer death in the United States by 2030, overtaking breast, prostate, and colorectal cancer (3). Most patients present with locally advanced or widely metastatic disease, rendering these tumors surgically unresectable (1–3). Even patients with localized tumors amenable to surgical resection will succumb to metastatic disease in almost all cases, suggesting that metastases occur prior to clinical presentation (1). Although previous studies identified mutant *KRAS* and molecular alterations inactivating *CDKN2A*, *TP53*, and TGF-β pathway components, these findings have not translated into improved therapies, nor have they led to effective screening strategies (3, 4, 5). Thus, there is a dire need to discover actionable mechanisms and new therapeutic targets for this exceptionally refractory tumor.

In contrast to many solid tumors, PDACs are characterized by a dense desmoplastic stroma composed of cancer-associated fibroblasts (CAFs) and fibrous scar tissue, although the role of the stroma in tumor progression remains controversial (6–11). While immune cells are found within the stroma, PDACs tend to be “cold” tumors, lacking an antitumor immune response (12). In vitro studies show that CAFs secrete factors that provide inflammatory signals and stimulate tumor growth and progression (9–11). Similarly, biomechanical analyses suggest that a “stiff” tumor microenvironment alters tumor cells to enhance motility and facilitate metastases (13–15). Further, PDAC cells grow faster when implanted with CAFs in mouse xenografts (16). The dense fibroblastic stroma also provides a barrier that prevents cytotoxic therapy from reaching tumor cells (9). Conversely, studies in transgenic mouse models of PDAC found that the stroma restrains tumor growth and progression (7, 8). More recent studies employing single-cell sequencing revealed that stromal cells, like cancer cells, are heterogeneous and impart tumor heterogeneity by creating various interfaces for tumor cells within their microenvironment (9, 17–23). These studies reveal a complex and nuanced role for the PDAC stroma, underscoring the need to better understand its role in disease progression.

Epigenetic alterations have emerged as a fundamental hallmark of cancer that drive tumorigenesis by altering cell fate decisions and differentiation (24). For example, genetic lesions involving the switch/sucrose nonfermentable (SWI/SNF) nucleosome remodeling complex occur in up to 15% of PDAC (25). Mutations affecting histone methyltransferase genes (mixed-lineage leukemia 2 and 3) and the gene encoding the histone demethylase lysine demethylase 6A (*KDM6A*), also arise in PDAC (25). Accordingly, aberrant methylation patterns are characteristic of PDAC (26–28). Genetic alterations that decrease sirtuin 6 (SIRT6) protein levels, a nutrient sensor and histone deacetylase that removes acetyl groups from histone 3 lysine 9 (H3K9) and histone 3 lysine 56 (H3K56), drive pancreatic tumorigenesis in murine models and predict a subclass of human PDAC with decreased survival (29). Although these discoveries shed light on epigenetic abnormalities in PDAC, they have not led to better therapies.

Overexpression of the gene encoding the chromatin regulator HMGA1 occurs in most aggressive tumors, including PDAC, where high levels portend poor differentiation and adverse outcomes (30–50). The *HMGA1* gene is normally expressed during embryogenesis (30, 39, 51) and in adult stem cells (46, 49, 52), but silenced postnatally in most differentiated cells. Through alternatively spliced mRNA, *HMGA1* encodes HMGA1a and HMGA1b isoforms, which bind to AT-rich sequences, bend chromatin, and recruit transcriptional complexes to modulate gene expression (31–35, 37, 39, 42, 45–47, 49, 53). When overexpressed in lymphoid cells of transgenic mice, *Hmga1* induces aggressive leukemia by upregulating transcriptional networks active in proliferating stem cells, poorly differentiated cancer cells, and inflammation (32, 35, 43, 47, 53). While mechanisms driving *HMGA1* expression in cancer are incompletely understood, growth factors (54, 55), cancer-associated mutations, including *Kras* (56) or mutant *Apc* (57), and oncogenic transcription factors, such as cMYC (58–60), upregulate *HMGA1*, suggesting that diverse oncogenic pathways converge on *HMGA1* to induce its expression. HMGA1 also cooperates with KRAS in immortalized pancreatic ductal epithelial cells to foster clonogenicity (61), whereas silencing *HMGA1* in PDAC cell lines disrupts metastatic progression following orthotopic implantation in immunodeficient mice (62). In intestinal stem cells, HMGA1 amplifies Wnt signals from the stroma and epithelial niches by inducing the expression of genes encoding Wnt agonist receptors (*Fzd5/7*, *Lrp5/6*, and *Lgr5*) and Wnt effectors, such as *cMyc* and *Sox9* (46). Together, these findings suggest that HMGA1 fosters tumor progression through both cell-intrinsic and stromal interactions, though little is known about transcriptional networks and tumor-stroma crosstalk governed by HMGA1 in PDAC.

Here, we uncover what we believe is a previously unknown epigenetic program whereby HMGA1 upregulates transcriptional networks involved in proliferation and tumor-stroma interactions during tumor progression and development of a fibroblastic stroma in PDAC. HMGA1 binds directly to the fibroblast growth factor 19 (*FGF19*) promoter and recruits active histone marks to induce FGF19 expression and secretion from PDAC cells. Silencing either *HMGA1* or *FGF19* disrupts phenotypes required for tumor progression. Surprisingly, loss of just a single *Hmga1* allele within the pancreatic ductal epithelium significantly prolongs survival in *Kras^+/LSL-G12D^; Trp53^+LSL-R172H^; Pdx1-Cre* (KPC) (63) mice compared with those with both *Hmga1* alleles intact. In mice with human PDAC xenografts, silencing *HMGA1* or *FGF19* depletes tumor-initiating cells while disrupting tumor growth and stroma formation. Moreover, treatment with an FGF receptor 4 (FGFR4) inhibitor, BLU9931, to block FGF19 function (64) recapitulates the effects of *HMGA1* or *FGF19* silencing, decreasing tumor growth and stroma formation in orthotopic models. Importantly, high expression of both *HMGA1* and *FGF19* defines a subclass of human PDAC with exceptionally poor outcomes. Together, our findings reveal a unique role for HMGA1 in tumor progression and “building” a stromal wall through FGF19 and highlight a new therapeutic target for a subset of highly recalcitrant tumors.

## Results

### Silencing HMGA1 disrupts oncogenic properties and depletes tumor-initiating cells.

**Figure 1:**
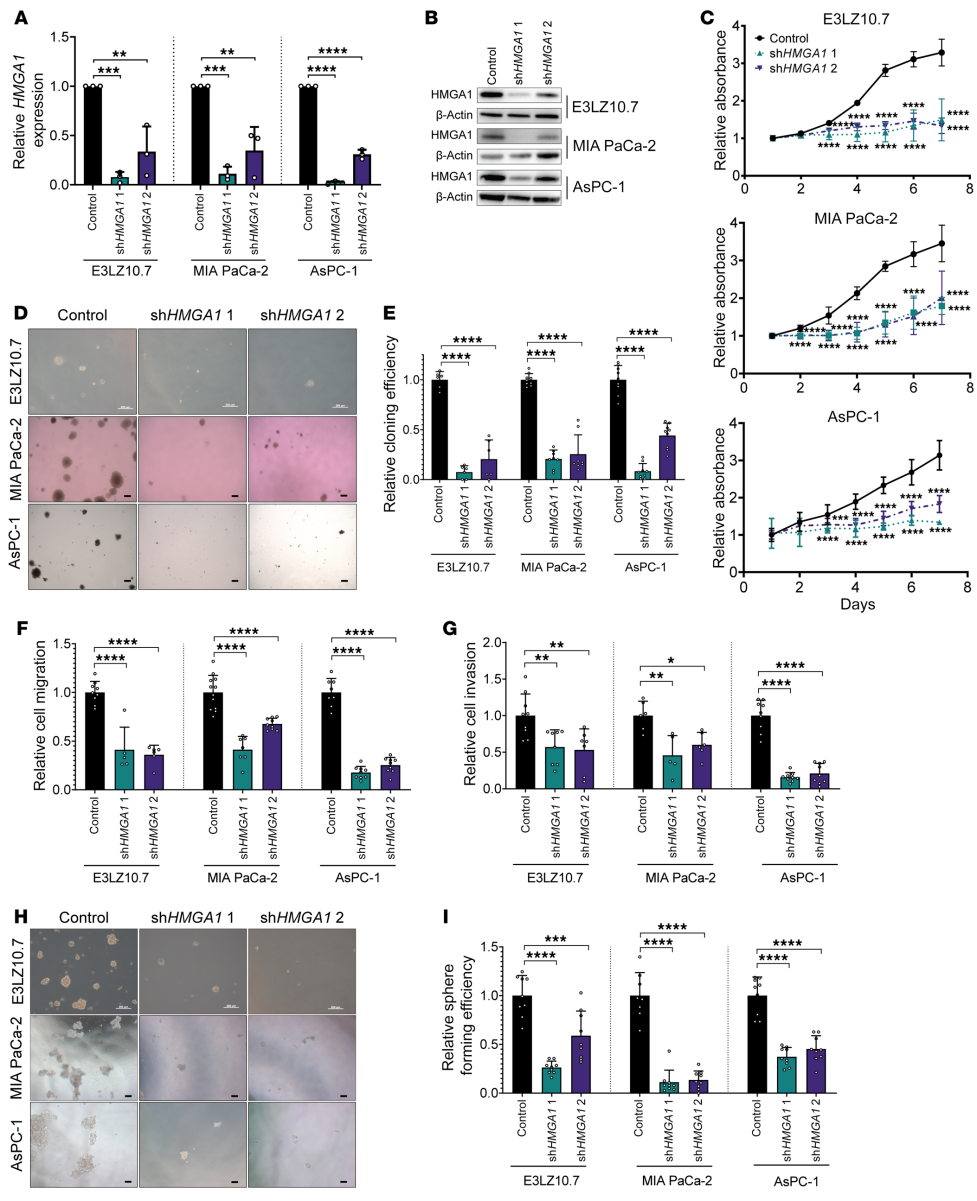
*HMGA1* knockdown disrupts oncogenic properties in PDAC cell lines. (**A**) *HMGA1* expression in PDAC cell lines (E3LZ10.7, MIA PaCa-2, AsPC-1) comparing controls (transduced with empty lentiviral vector) to *HMGA1* silencing via lentiviral delivery of shRNA targeting 2 different sequences (sh*HMGA1* 1, sh*HMGA1* 2) from 3 experiments performed in triplicate. (**B**) Representative immunoblots (*n* = 3 experiments) of HMGA1 in PDAC cells with and without *HMGA1* silencing. (**C**) Proliferation (by MTT) comparing PDAC cells with and without *HMGA1* silencing from 3 experiments performed in triplicate. (**D**) Representative images of soft agar clonogenicity assay in PDAC cells with and without *HMGA1* silencing (E3LZ10.7, *n* = 2; MIA PaCa-2 and AsPC-1, *n* = 3). Scale bars: 200 μm. (**E**) Clonogenic efficiency comparing PDAC cell lines with and without *HMGA1* silencing from experiments performed in triplicate (E3LZ10.7, *n* = 2; MIA PaCa-2 and AsPC-1, *n* = 3). (**F**) Migration comparing PDAC cells with and without *HMGA1* silencing following treatment with 10 μM cytosine β-D-arabinoside (AraC) for 1 hour to mitigate effects of proliferation from experiments performed in triplicate (E3LZ10.7 and MIA PaCa-2, *n* = 2; AsPC-1, *n* = 3). (**G**) Invasion comparing PDAC cells with and without *HMGA1* silencing following treatment with 10 μM AraC for 1 hour to mitigate effects of proliferation from experiments performed in triplicate (MIA PaCa-2, *n* = 2; E3LZ10.7 and AsPC-1, *n* = 3). (**H**) Representative images (*n* = 3 experiments) of 3D sphere formation in PDAC cell lines with and without *HMGA1* silencing. Scale bars: 200 μm. (**I**) 3D sphere formation comparing PDAC cell lines with and without *HMGA1* silencing from 3 experiments performed in triplicate. Data are presented as mean ± standard deviation (SD). *P < 0.05, **P < 0.01, ***P < 0.001, ****P < 0.0001 by 1-way ANOVA with Dunnett’s multiple-comparison test (**A**, **C**, **E**–**G**, and **I**). Scale bars: 200 μm.

Because *HMGA1* is upregulated in PDACs where high levels associate with decreased survival (36, 38, 61, 62), we sought to elucidate *HMGA1* function in pancreatic carcinogenesis. First, we found that HMGA1 expression (mRNA and protein) is higher in PDAC cell lines derived from metastatic tumors compared with those from primary tumors (65) (Supplemental Figure 1, A–E; supplemental material available online with this article; https://doi.org/10.1172/JCI151601DS1). Next, we silenced *HMGA1* via lentiviral delivery of short hairpin RNAs (shRNAs) targeting 2 different sequences (49) in cell lines from primary and metastatic tumors harboring common PDAC mutations: (a) E3LZ10.7 (66), from a liver metastasis with *KRAS^G12D^* and homozygous SMAD4 deletion; (b) MIA PaCa-2 (67), from a primary PDAC with homozygous *CDKN2A/p16^INK4A^* deletion, mutant *KRAS^G12C^*, and *TP53*; and (c) AsPC-1 (67), from PDAC ascites fluid with homozygous mutations in *KRAS^G12D^*, TP53^C135fs*35^, and CDKN2A^L78fs*41^. Strikingly, HMGA1 deficiency disrupted proliferation, clonogenicity, migration, invasion, and 3-dimensional (3D) sphere formation in all cell lines tested (Figure 1), indicating that HMGA1 is required for these oncogenic properties.

**Figure 2:**
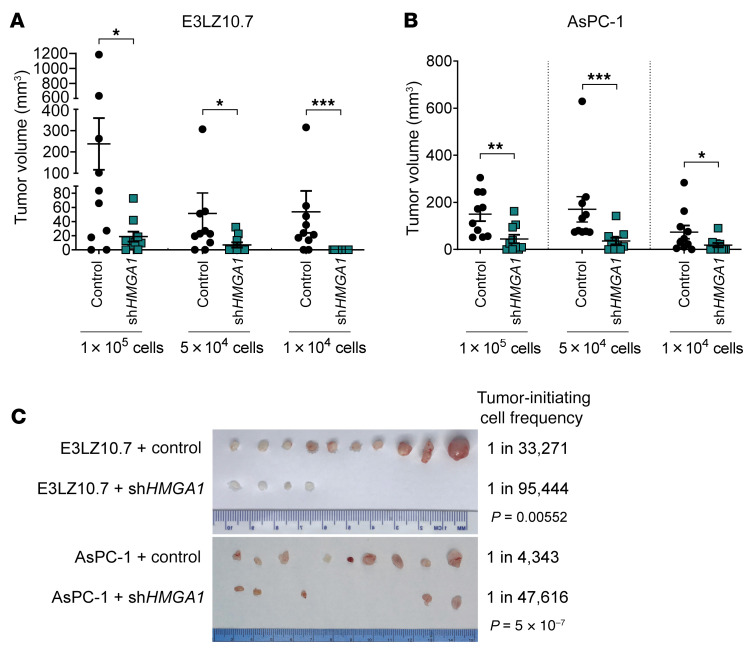
*HMGA1* knockdown disrupts tumorigenesis and depletes tumor-initiating cells. (**A**) Xenograft tumorigenicity at limiting dilutions comparing E3LZ10.7 cell *HMGA1* silencing (*n* = 10/condition). (**B**) Xenograft tumorigenicity at limiting dilutions comparing AsPC-1 cells with and without *HMGA1* silencing (*n* = 10/condition). (**C**) Comparison of tumors dissected at the completion of experiment with 1 × 10^4^ PDAC cells with and without *HMGA1* silencing (left) and calculated frequency of tumor-initiating cells (right) in PDAC cells (E3LZ10.7, AsPC-1) with and without *HMGA1* silencing. Tumor-initiating cell frequency was calculated by extreme limiting dilution analysis (ELDA; ref. 102). Data shown as mean ± standard error of the mean (SEM). *P < 0.05, **P < 0.01, ***P < 0.001 by Mann-Whitney test (**A** and **B**) or χ^2^ test (**C**).

To define HMGA1 function in vivo, we assessed xenograft tumorigenesis from PDAC cell lines (E3LZ10.7 and AsPC-1), which showed that HMGA1 deficiency decreases tumor volumes (Figure 2, A and B). Intriguingly, tumors that formed from the pool of cells with *HMGA1* silencing (E3LZ10.7 and AsPC-1 cells) express higher *HMGA1* than the injected cells, suggesting that escape from gene silencing and a specific level of HMGA1 is required for tumor formation (Supplemental Figure 1, F and G). HMGA1 deficiency also depletes tumor-initiating cells in both cell lines (E3LZ10.7 and AsPC-1), demonstrating that HMGA1 is required for tumor initiation and growth in xenograft models (Figure 2C and Supplemental Figure 1, H and I).

### HMGA1 regulates transcriptional networks involved in proliferation and signaling.

**Figure 3:**
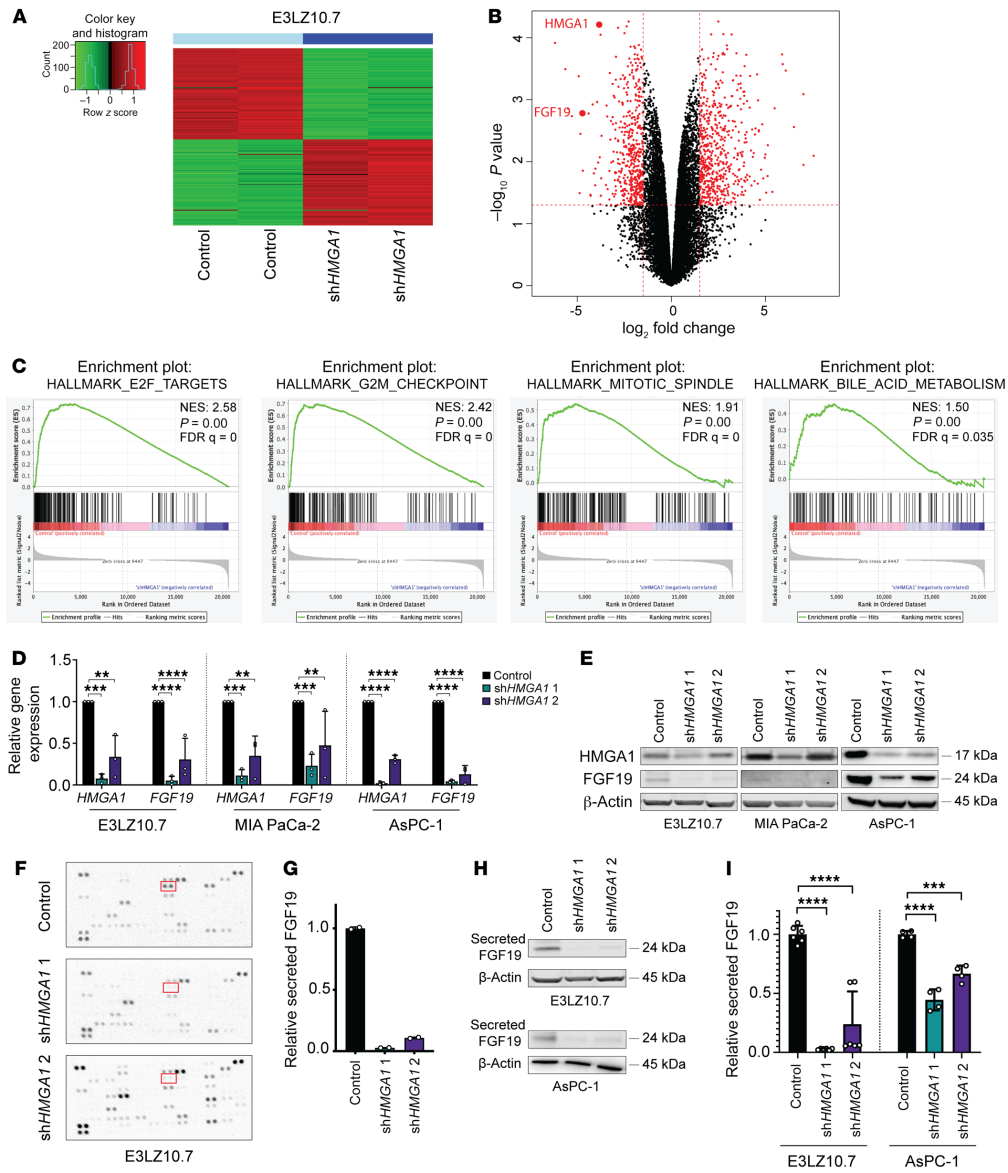
HMGA1 induces FGF19 expression and secretion in PDAC cell lines. (**A**) Heatmap from hierarchical, supervised clustering of differentially expressed genes (DEGs) comparing control E3LZ10.7 cells to those with *HMGA1* silencing (performed in duplicate in 1 RNA sequencing experiment). (**B**) Volcano plot of DEGs in E3LZ10.7 with and without *HMGA1* silencing reveals *FGF19* among the genes most repressed with *HMGA1* silencing. Thresholds are shown as dashed red lines; genes (dots) with significant differential expression are shown in red. *P* < 0.05, log_2_(fold change) > 1.5. (**C**) GSEA of DEGs induced by HMGA1 in E3LZ10.7 controls (high HMGA1) compared to those with *HMGA1* silencing show enrichment for gene sets associated with proliferation (E2F targets, G_2_/M checkpoint, mitotic spindle) and bile acid metabolism (MSigDb Hallmark). Normalized enrichment score (NES), false discovery rate (FDR), and *P* values are shown. (**D**) *FGF19* expression in PDAC cells (E3LZ10.7, MIA PaCa-2, AsPC-1) with and without *HMGA1* silencing from 3 experiments performed in triplicate. (**E**) Representative immunoblots (*n* = 3 experiments) of FGF19 levels in PDAC cells with and without *HMGA1* silencing. (**F**) Cytokine arrays of secreted protein in E3LZ10.7 cells when *HMGA1* is silenced. (**G**) Secreted FGF19 (relative pixel density) of duplicate spots on a single cytokine array per condition (control versus *HMGA1* silencing via sh*HMGA1* 1 or sh*HMGA1* 2). (**H**) Representative immunoblots (*n* = 3 experiments) of secreted FGF19 in PDAC cells (E3LZ10.7, AsPC-1) with and without *HMGA1* silencing. (**I**) Secreted FGF19 comparing PDAC cells (E3LZ10.7, AsPC-1) with and without *HMGA1* silencing by ELISA from experiments performed in duplicate (E3LZ10.7, *n* = 3; AsPC-1, *n* = 2). Data are presented as mean ± SD. **P < 0.01, ***P < 0.001, ****P < 0.0001 by 1-way ANOVA with Dunnett’s multiple-comparison test (**D** and **I**).

To identify HMGA1 transcriptional networks, we performed RNA sequencing (GSE222890) in E3LZ10.7 cells (Figure 3, A and B) with or without *HMGA1* silencing. Unsupervised hierarchical clustering separated cells with high *HMGA1* (controls) from those with *HMGA1* silencing (Supplemental Figure 2A). Differentially expressed genes (*P* < 0.05, log_2_[fold change] > 1.5) (68) included 660 up- and 565 downregulated genes (Figure 3B). Gene set enrichment analysis (GSEA, MSigDb Hallmark gene sets) revealed an HMGA1 signature of genes involved in cell cycle progression (E2F targets, G_2_/M checkpoint, mitotic spindle genes) (Figure 3C), while curated gene sets showed enrichment for cell cycle progression, cell signaling, metastatic progression, cancer stem cells, and embryonic stem cells (Supplemental Table 1) (69, 70). Unexpectedly, we identified gene sets associated with bile acid metabolism, a pathway regulated, in part, by FGF19. Intriguingly, *FGF19* (Figure 3B) was among the genes most robustly upregulated by HMGA1, with greater than 20-fold differential expression. Given this robust upregulation and because growth factors can function in cell-autonomous and tumor-stroma interactions, we focused on *FGF19* first. In other contexts, *FGF19* promotes proliferation, and *Fgf15*, the murine homolog, induces hepatocellular carcinogenesis and fibrosis in mice (71–74). Further, clinical inhibitors are available to target FGF19 or its receptor, FGFR4 (64, 75–77), although the role of FGF19 in pancreatic carcinogenesis is unknown.

### HMGA1 induces FGF19 expression and secretion.

HMGA1-dependent expression of FGF19 (mRNA, protein) was validated in PDAC cell lines (E3LZ10.7, MIA PaCa-2, and AsPC-1; Figure 3, D and E). Intriguingly, *FGF19* levels were much higher in the metastatic cell lines (E3LZ10.7 and AsPC-1) compared with MIA PaCa-2 cells derived from a localized tumor (Supplemental Figure 2B). Because FGF19 protein was barely detectable in MIA PaCa-2 cells, we validated its HMGA1 dependence by immunoprecipitation (IP) (Supplemental Figure 2C). Because FGF19 is secreted from cells and could function in an autocrine and/or paracrine fashion, we assessed secretion from E3LZ10.7 cells by cytokine arrays, which show a marked decrease with *HMGA1* silencing (Figure 3, F and G); these results were validated by immunoblotting and ELISA of media (Figure 3, Hand I). Six additional secreted factors were repressed with *HMGA1* silencing, 7 were increased, and 9 were unchanged (Supplemental Figure 2, D–F). Similar to the gene expression results, secreted FGF19 was among the most robustly repressed factors with HMGA1 deficiency. FGF19 secretion from AsPC-1 or MIA-PaCa-2 cells also decreased with *HMGA1* silencing, as detected by ELISA of media (Figure 3I and Supplemental Figure 2G). Together, these results demonstrate that FGF19 gene expression, protein levels within PDAC cells, and secretion depend upon HMGA1 in E3LZ10.7, MIA PaCa-2, and AsPC-1 cell lines.

### Silencing FGF19 recapitulates effects of silencing HMGA1.

**Figure 4:**
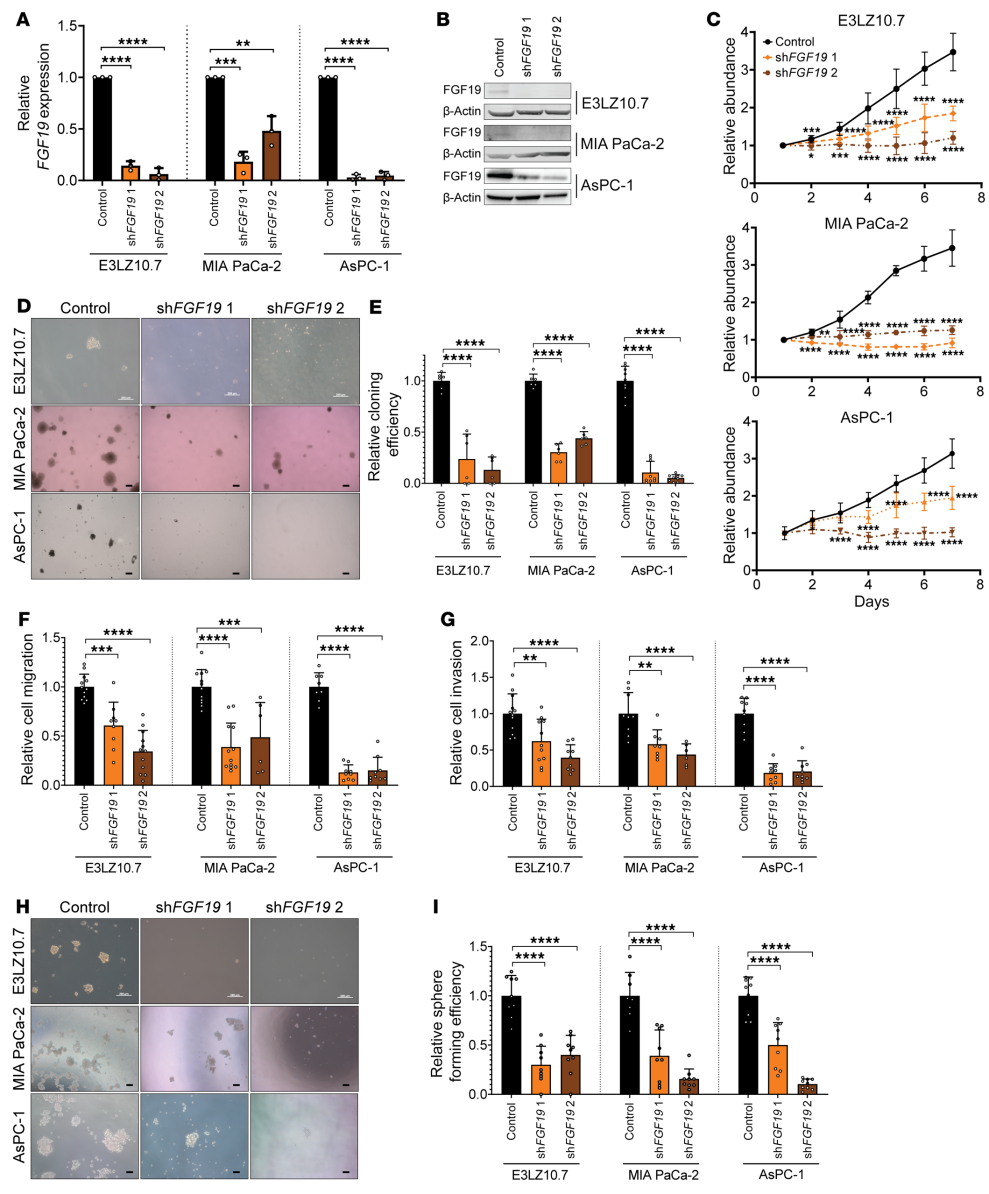
*FGF19* knockdown recapitulates most phenotypes associated with HMGA1 deficiency in PDAC cell lines. (**A**) *FGF19* expression in PDAC cells (E3LZ10.7, MIA PaCa-2, AsPC-1) comparing controls (empty lentiviral vector) to those with *FGF19* silencing via lentiviral delivery of shRNA targeting 2 different sequences (sh*FGF19* 1, sh*FGF19* 2) from 3 experiments performed in triplicate. (**B**) Representative immunoblots (*n* = 3 experiments) of FGF19 protein levels in PDAC cells with and without *FGF19* silencing. (**C**) MTT proliferation assays comparing PDAC cells with and without *FGF19* silencing from 2 experiments performed in triplicate. (**D**) Representative images of clonogenicity assay comparing PDAC cells with and without *FGF19* silencing (E3LZ10.7, MIA PaCa-2, *n* = 2; AsPC-1, *n* = 3). Scale bars: 200 μm. (**E**) Clonogenic efficiency comparing PDAC cell lines with and without *HMGA1* silencing from experiments performed in triplicate (E3LZ10.7, MIA PaCa-2, *n* = 2; AsPC-1, *n* = 3). (**F**) Migration assay comparing PDAC cells with and without *FGF19* silencing following treatment with 10 μM β-D-arabinoside (AraC) for 1 hour to mitigate effects of proliferation silencing from experiments performed in triplicate (MIA PaCa-2, *n* = 2; E3LZ10.7, AsPC-1, *n* = 3). (**G**) Invasion assay comparing PDAC cells with and without *FGF19* silencing following treatment with 10 μM AraC for 1 hour to mitigate effects of proliferation silencing from experiments performed in triplicate (MIA PaCa-2, *n* = 2; E3LZ10.7, AsPC-1, *n* = 3). Scale bars: 200 μm. (**H**) Representative images (*n* = 3 experiments) of 3D sphere-formation assay comparing PDAC cells with and without *HMGA1* silencing. (**I**) 3D sphere formation comparing PDAC cell lines with and without *HMGA1* silencing from 3 experiments performed in triplicate. Data are presented as mean ± SD. *P < 0.05, **P < 0.01, ***P < 0.001, ****P < 0.0001 by 1-way ANOVA with Dunnett’s multiple-comparison test (**A**, **C**, **E**–**G**, and **I**). Scale bars: 200 μm.

**Figure 5:**
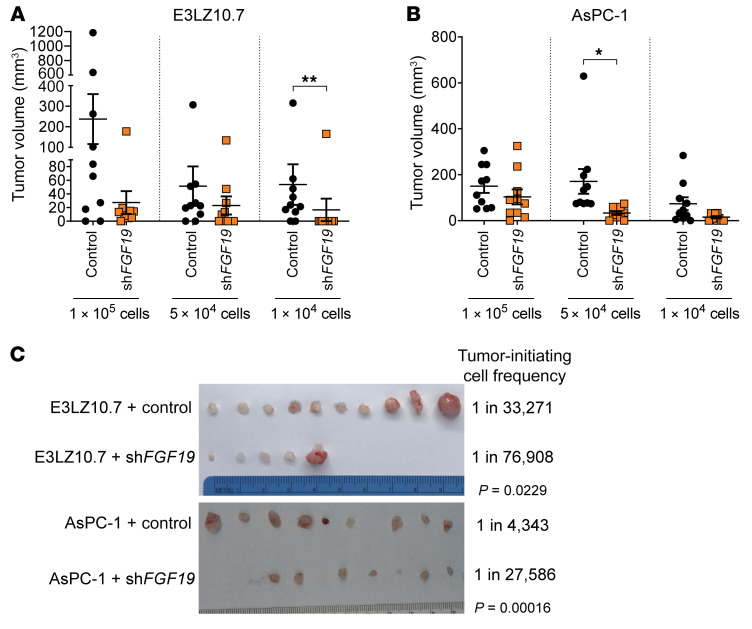
*FGF19* knockdown disrupts tumorigenesis and depletes tumor-initiating cells, similar to phenotypes observed with *HMGA1* silencing in PDAC xenografts. (**A**) Xenograft tumorigenicity at limiting dilutions comparing E3LZ10.7 cells with and without *FGF19* silencing (*n* = 10/condition). (**B**) Xenograft tumorigenicity at limiting dilutions comparing AsPC-1 cells with and without *FGF19* silencing (*n* = 10/condition). (**C**) Comparison of tumors dissected at the completion of experiment with 1 × 10^4^ PDAC cells (E3LZ10.7, AsPC-1) with and without *FGF19* silencing (left) and calculated frequency of tumor-initiating cells (right) among PDAC cells. Tumor-initiating cell frequency calculated by ELDA (102). Data shown as mean ± SEM. *P < 0.05, **P < 0.01 by Mann-Whitney test (**A** and **B**) or χ^2^ test (**C**).

To determine whether FGF19 is required for HMGA1 function in PDAC, we silenced *FGF19* in PDAC cell lines (E3LZ10.7, MIA PaCa-2, and AsPC-1) via lentiviral delivery of shRNAs targeting 2 different sequences (Figure 4, A and B, and Supplemental Figure 3A). Surprisingly, silencing *FGF19* faithfully recapitulated phenotypes observed with HMGA1 deficiency, disrupting proliferation, colony formation, migration, invasion, and 3D sphere formation (Figure 4, C–I). As an alternative approach to inhibit FGF19, we tested BLU9931, an inhibitor that specifically blocks the canonical FGF19 receptor (FGFR4) (64), demonstrating that BLU9931 impairs the proliferation, migration, and invasiveness of PDAC cell lines (E3LZ10.7 and MIA PaCa-2; Supplemental Figure 3, B–D). In xenograft tumorigenesis with E3LZ10.7 and AsPC-1 cells, both of which express higher levels of FGF19, the knockdown of *FGF19* decreased tumor volumes and tumor-initiating cells (Figure 5, A–C, and Supplemental Figure 3, E and F). Intriguingly, in *FGF19*-silenced tumors, one E3LZ10.7 tumor at each dilution and one AsPC-1 tumor at the lowest dilution grew to proportions equal to or greater than controls. We therefore reassessed *FGF19* levels in these tumors and noted a marked increase in *FGF19* relative to the injected pool, suggesting that escape from *FGF19* silencing allowed enhanced tumor growth (Supplemental Figure 3, G and H).

To determine whether exogenous FGF19 could rescue the effects of *HMGA1* silencing, we exposed PDAC cells with *HMGA1* silencing (E3LZ10.7) to recombinant human FGF19 (hFGF19). Proliferation (via 5-ethynyl-2′-deoxyuridine [EdU] incorporation) increased upon treatment with hFGF19, but not to levels of the control cells (Supplemental Figure 3I), indicating that FGF19 is required, but not sufficient, for proliferation mediated by HMGA1. Together, our results indicate that *FGF19* is a partial mediator of HMGA1 oncogenic function in these PDAC models.

### HMGA1 binds directly to the FGF19 promoter and recruits activating histone marks.

**Figure 6:**
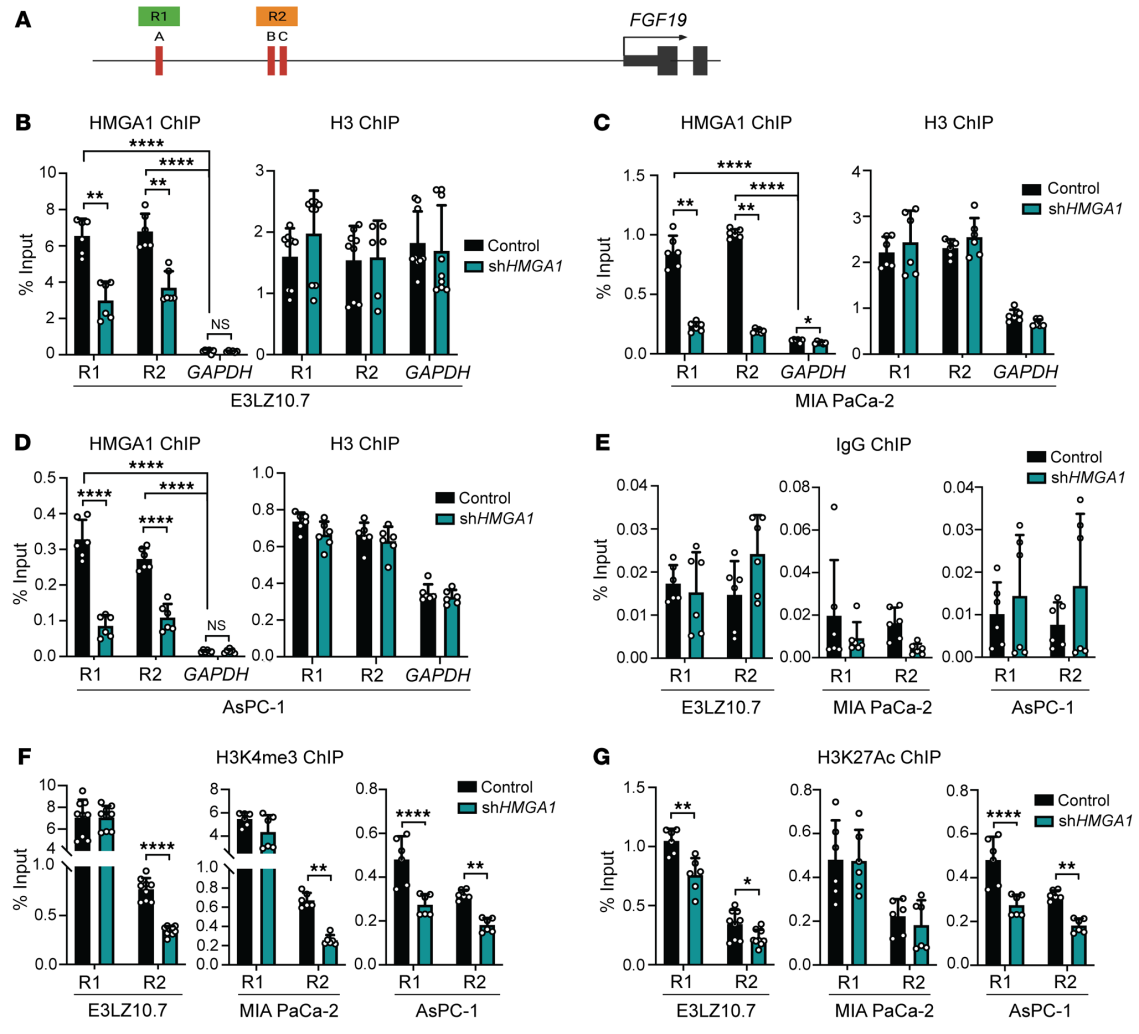
HMGA1 induces *FGF19* expression by binding to the *FGF19* promoter and recruiting active histone marks. (**A**) Schematic representation of the *FGF19* promoter; R1 includes predicted HMGA1 binding site A; R2 includes predicted HMGA1 sites B and C. (**B**) ChIP-PCR comparing HMGA1 occupancy on the *FGF19* promoter in E3LZ10.7 cells with and without *HMGA1* silencing. (**C**) ChIP-PCR comparing HMGA1 occupancy on the *FGF19* promoter in MIA PaCa-2 cells with and without *HMGA1* silencing. (**D**) ChIP-PCR comparing HMGA1 occupancy on the *FGF19* promoter in AsPC-1 cells with and without *HMGA1* silencing. In **B**–**D**, histone H3 served as a positive control for chromatin pull-down and the *GAPDH* promoter sequence as a negative control. (**E**) ChIP-PCR of control IgG at R1 and R2 in PDAC cells (E3LZ10.7, MIA PaCa-2, AsPC-1) with and without *HMGA1* silencing. (**F**) ChIP-PCR for the H3K4me3 active histone mark on the *FGF19* promoter in PDAC cells (E3LZ10.7, MIA PaCa-2, AsPC-1) with and without *HMGA1* silencing. (**G**) ChIP-PCR for the H3K27Ac active histone mark on the *FGF19* promoter in PDAC cells with and without *HMGA1* silencing. All ChIP-PCR results are shown from 2 experiments performed in triplicate. Data are presented as mean ± SD. Significance was evaluated by 1-way ANOVA with Dunnett’s multiple-comparison test (**B**–**E**), 2-tailed Student’s *t* test (E3LZ10.7, AsPC-1 cells; data normally distributed) and Mann-Whitney test (MIA Paca-2 cells; data not normally distributed) (**F**), or Mann-Whitney test (**G**). *P < 0.05, **P < 0.01, ****P < 0.0001.

Using an in silico prediction algorithm (MatInspector) (78), we identified putative HMGA1 DNA binding sites within the *FGF19* promoter at –1092, –832, and –810 base pairs (designated sites A, B, and C, respectively) upstream of the transcription start site (TSS) (Figure 6A). HMGA1 occupancy by chromatin IP–PCR (ChIP-PCR) demonstrated that regions (~200 base pairs) surrounding site A (region 1, R1) or the region encompassing sites B and C (R2) show enrichment for HMGA in cell lines (E3LZ10.7, MIA PaCa-2, and AsPC-1), which was depleted with *HMGA1* knockdown (Figure 6, B–D). The positive control, histone H3, was unchanged with HMGA1 deficiency. By contrast, there was no significant occupancy, nor were there changes with HMGA1 deficiency using a negative control IgG antibody (Figure 6E).

Because our gene expression data show that HMGA1 induces *FGF19*, we assessed occupancy of active histone H3 lysine 4 trimethylation (H3K4me3) and histone H3 lysine 27 acetylation (H3K27Ac), both of which mark promoter and enhancer regions. In 3 cell lines (E3LZ10.7, MIA PaCa-2, and AsPC-1), H3K4me3 was abundant at R1 and R2 and decreased with *HMGA1* silencing (Figure 6, E–G). HMGA1 deficiency also depleted H3K4me3 at R1 in AsPC-1 cells (Figure 6G). These data indicate that HMGA1 binds directly to the *FGF19* promoter at R1 and R2 and recruits H3K4me3 in all 3 PDAC cell lines. In the metastatic E3LZ10.7 and AsPC-1 cell lines, HMGA1 also recruited H3K27Ac to R2. Of note, H3K27Ac histone marks associate with poised chromatin, stretch, or “super-enhancers,” and regulation of developmental or stem cell–like genes during normal development and in cancer (79). Poised enhancers at developmental promoters are also implicated in poorly differentiated cancers and cancer stem cells (80). Although there are differences in the specific histone marks between cell lines, HMGA1 was consistently associated with occupancy of active histone marks at the *FGF19* promoter in all 3 cell lines.

**Figure 7:**
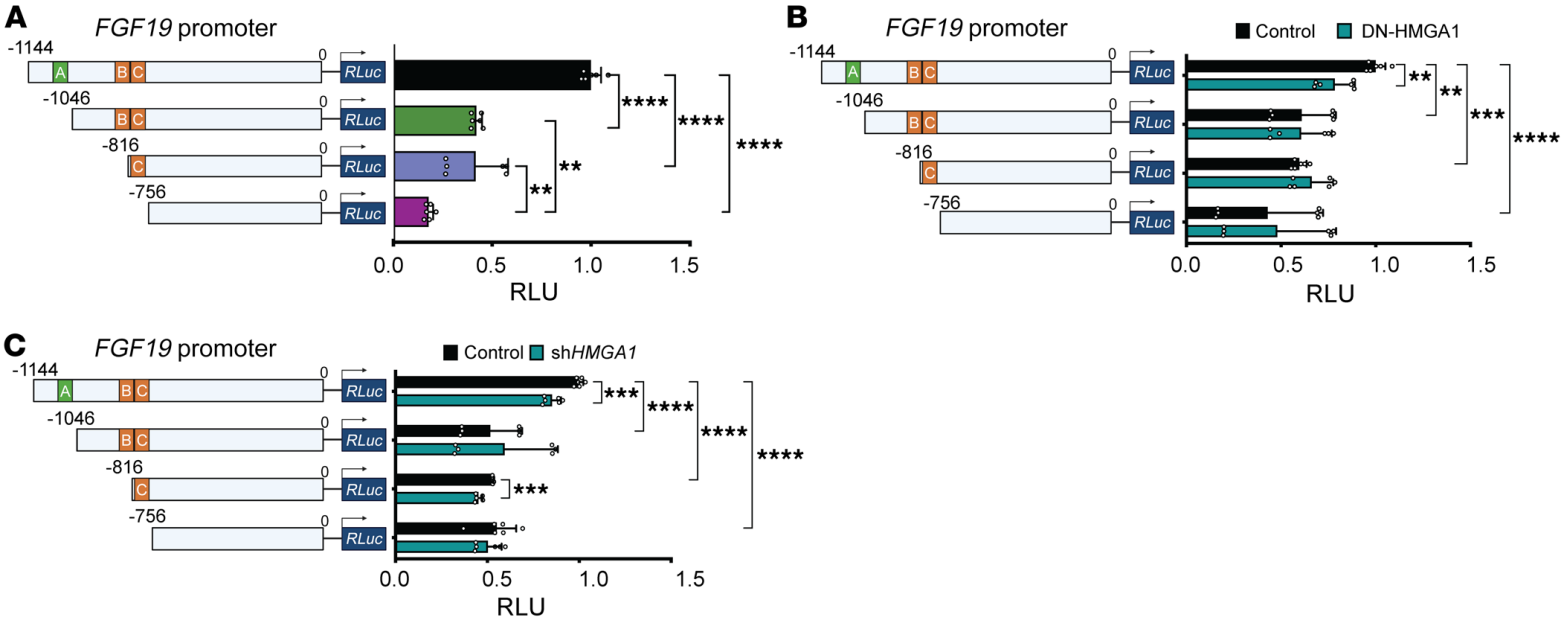
HMGA1 binds to the *FGF19* promoter to induce *FGF19* expression. (**A**) Reporter gene activity (via dual-luciferase assay) in E3LZ10.7 cells transfected with *FGF19* promoter constructs. (**B**) Reporter gene activity (via dual-luciferase assay) in E3LZ10.7 cells after cotransfection with dominant-negative HMGA1 or control vector and *FGF19* promoter constructs. (**C**) Reporter gene activity (via dual-luciferase assay) in E3LZ10.7 cells after cotransfection with *HMGA1* silencing or control vector and *FGF19* promoter constructs. Data shown as mean ± SD from 2 independent experiments performed in triplicate. **P < 0.01, ***P < 0.001, ****P < 0.0001 by 1-way ANOVA with Dunnett’s multiple-comparison test (**A**–**C**). RLU, relative luminescence units.

To functionally validate these chromatin marks, we determined whether HMGA1 transactivates the *FGF19* promoter linked to a luciferase reporter gene. We tested a promoter construct (–1144) including regions R1, R2, and downstream sequences up to the TSS compared with constructs with 5′ deletions: (a) –1046, lacking R1 and site A; (b) –816, lacking R1, site A, 5′ sequences of R2, and site B; and (c) –756, lacking R1, R2, and sites A, B, and C. As expected, the –1144 construct showed the greatest reporter activation, with decreases in constructs –816 and –756, and the lowest activity in the construct lacking both R1 and R2 (Figure 7A). Promoter activity of the full-length construct also decreases to levels of the deletion constructs in the presence of either a dominant-negative HMGA1 that no longer binds to DNA (81) or with *HMGA1* silencing (Figure 7, B and C). These findings indicate that HMGA1 directly transactivates *FGF19* expression by binding to R1 and R2 and recruiting active histone marks.

### HMGA1 signals through FGF19/FGFR4.

**Figure 8:**
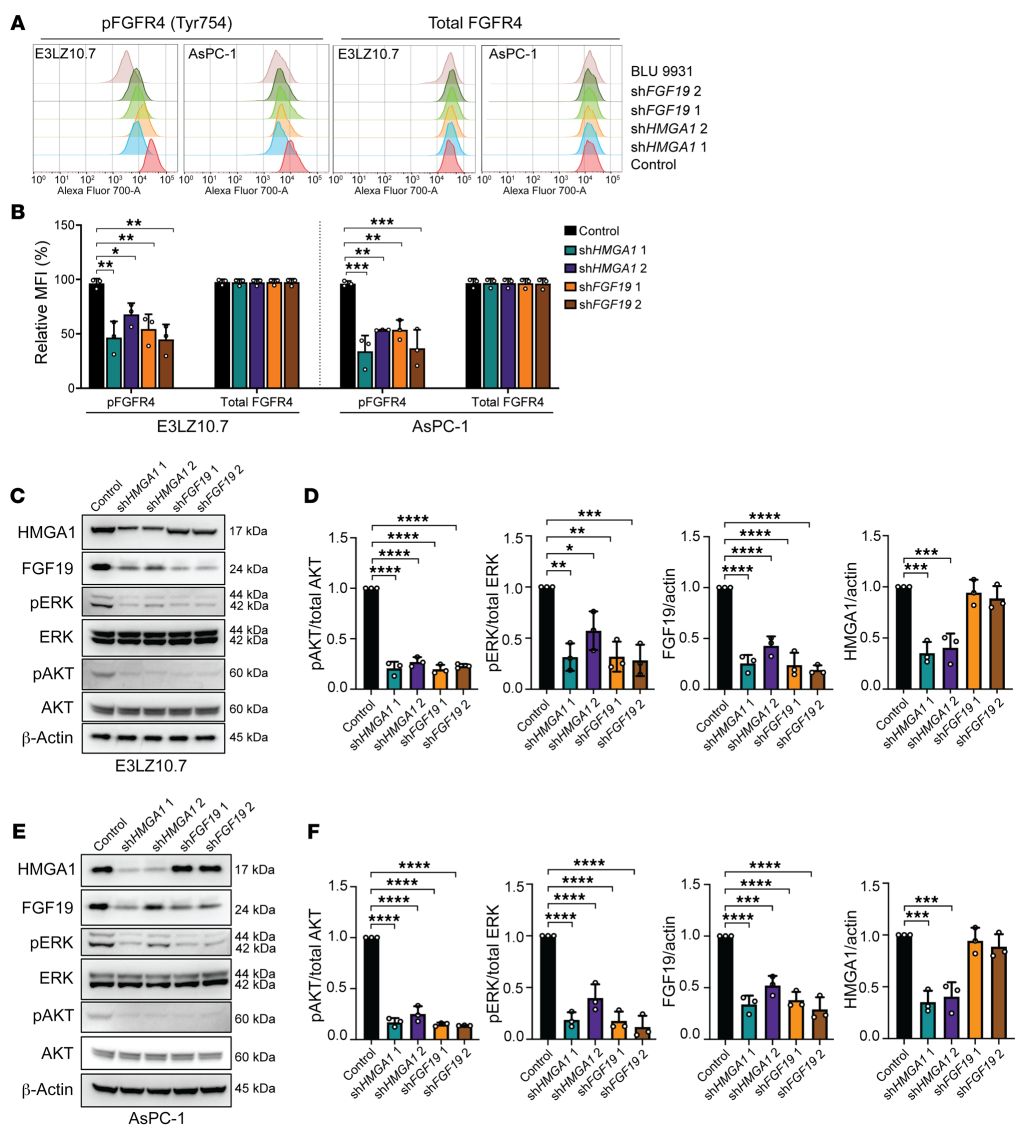
HMGA1 signals through the canonical FGF19/FGFR4 pathway. (**A**) Representative flow cytometric profiles (*n* = 3 experiments) of phosphorylated FGFR4 (p-FGFR4) and total FGFR4 in PDAC cell lines (E3LZ10.7, AsPC-1) with and without *HMGA1* silencing, *FGF19* silencing, or treatment with the FGFR4 inhibitor BLU9931 (10 μM). (**B**) Comparison of mean fluorescence intensities (MFIs) of phosphorylated FGFR4 (p-FGFR4) and total FGFR4 in PDAC cell lines (E3LZ10.7, AsPC-1) with and without *HMGA1* silencing, *FGF19* silencing, or treatment with BLU9931 (10 μM). (**C**) Representative immunoblots (*n* = 3 experiments) and (**D**) relative protein levels of FGFR4 and downstream signaling molecules (ERK, AKT), including total protein and phosphorylated proteins in E3LZ10.7 cells with and without *HMGA1* or *FGF19* silencing. (**E**) Representative immunoblots (*n* = 3 experiments) and (**F**) relative protein levels of FGFR4 and downstream signaling molecules in AsPC-1 cells with and without *HMGA1* or *FGF19* silencing. Data shown as mean ± SD from 3 independent experiments performed in triplicate. *P < 0.05, **P < 0.01, ***P < 0.001, ****P < 0.0001 by 1-way ANOVA with Dunnett’s multiple-comparison test (**B**, **D**, and **F**).

To determine whether HMGA1 and FGF19 signal through FGFR4, we assessed phosphorylation of FGFR4 and downstream signals (ERK and AKT) by flow cytometry and Western blotting in PDAC cells (E3LZ10.7 and AsPC-1). Silencing either *HMGA1* or *FGF19* decreased phosphorylation of FGFR4 (p-FGFR4; by flow cytometry) and downstream signaling molecules (ERK and AKT) without affecting unphosphorylated protein levels, indicating that both HMGA1 and FGF19 transduce signals through canonical FGF19/FGFR4 pathways (Figure 8, A–F). After rendering cells (E3LZ10.7, MIA-PaCa-2, and AsPC-1) quiescent by serum deprivation, FGFR4 phosphorylation and proliferation increased with exposure to recombinant hFGF19 (Supplemental Figure 4, A–F). Together, these results suggest that HMGA1 induces *FGF19* expression and protein secretion, resulting in the phosphorylation of FGFR4 and downstream signaling molecules to enhance proliferation in PDAC cells.

### HMGA1 and FGF19 associate with fibrotic stroma formation.

**Figure 9:**
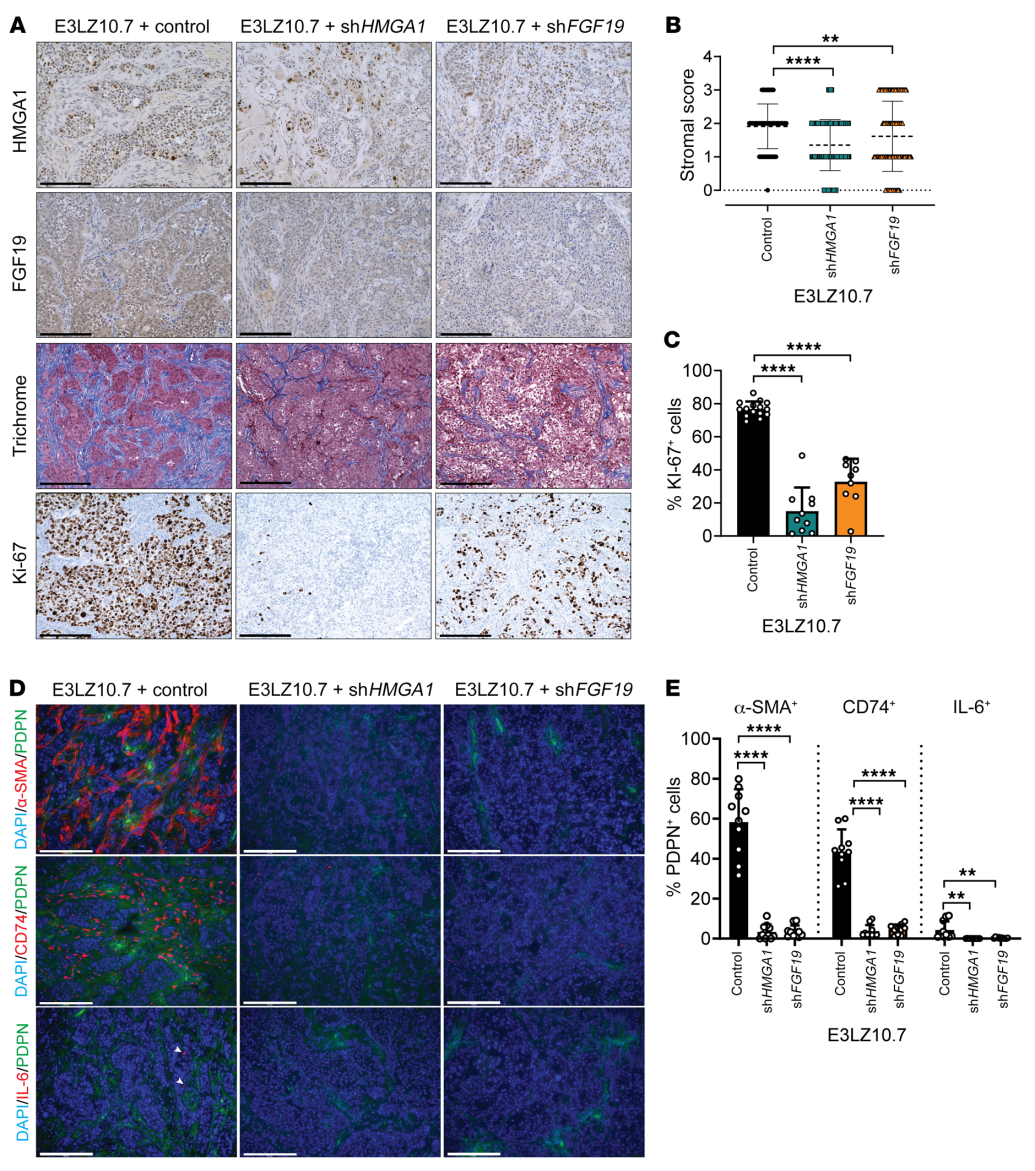
HMGA1 and FGF19 induce fibrotic stroma formation, proliferation (Ki-67), and modulate CAF composition during PDAC xenograft tumorigenesis. (**A**) Representative images (*n* = 10 per condition) of HMGA1 (IHC, top row), FGF19 (IHC, second row), fibrosis (trichrome, third row), and Ki-67 (IHC, bottom row) in E3LZ10.7 xenografts with and without *HMGA1* or *FGF19* silencing. (**B**) Quantitative comparison of stroma scores in E3LZ10.7 xenografts with and without *HMGA1* or *FGF19* silencing. Fibrosis scores based on 3-point system (0, <5%; 1, 5%–30%; 2, 30%–60%; 3, >60%) (*n* = 16 images taken from 3 control tumors, 2 sh*HMGA1* tumors, and 4 sh*FGF19* tumors). (**C**) Comparison of Ki-67–positive cell number in xenografts (5 fields at ×20 magnification of tumors from 2 different mice/group, with *n* = 10 per condition). (**D**) Representative IF images to compare CAF composition in E3LZ10.7 xenografts with and without *HMGA1* or *FGF19* silencing. (**E**) Total CAF numbers were ascertained by costaining with DAPI and for PDPN; α-SMA, CD74, and IL-6 were used to identity different subtypes of CAFs. Data in **D** and **E** were based on 10 fields at ×20 magnification (*n* = 10 per condition). Data presented as mean ± SD. **P < 0.01, ****P < 0.0001 by 1-way ANOVA with Dunnett’s multiple-comparison test (**B**, **C**, and **E**). Scale bars: 200 μm.

Because secreted FGF19 could interact with stroma, we determined whether HMGA1 or FGF19 modulates fibrosis (via trichrome staining) and CAF composition within the stroma. Fibrosis scores were assigned based on area staining with trichrome: 0 (<5%), 1 (5%–30%), 2 (30%–60%), and 3 (>60%). In control PDAC xenografts, extensive fibrosis comprised over 30%–60% of tumor volumes (fibrosis scores 2–3) and included both stromal cells with a characteristic fibroblast appearance (Figure 9, A and B, and Supplemental Figure 5, A and B) and tumor cells with extensive intranuclear HMGA1 staining and cytoplasmic FGF19 staining by immunohistochemistry (IHC) (Figure 9A and Supplemental Figure 5A). In contrast, xenografts from PDAC cells with *HMGA1* or *FGF19* silencing had less fibrosis (<30% area; Figure 9, A and B, and Supplemental Figure 5, A and B). Both HMGA1 and FGF19 staining also decreased in tumors from PDAC cells with *HMGA1* silencing and *FGF19* silencing also decreased FGF19 staining (Figure 9A and Supplemental Figure 5A). Of note, tumors arising from cells with *HMGA1* knockdown included a subset of tumor cells with HMGA1 intranuclear staining resembling controls, consistent with our gene expression data suggesting that escape from *HMGA1* silencing allows tumor cells to grow as xenografts (Supplemental Figure 1, F and G). Further, the proliferation marker Ki-67 decreased with *HMGA1* or *FGF19* silencing in PDAC xenografts (Figure 9, A and C, and Supplemental Figure 5, A and C). These findings indicate that HMGA1 and FGF19 promote tumor proliferation and stroma formation in xenografted tumors.

To elucidate HMGA1-dependent changes in CAF composition within the stroma of xenografted tumors, we performed immunofluorescence (IF) to classify CAFs into 3 major subtypes previously defined in KPC mice and human tumors (19–23) based on positive staining for podoplanin (PDPN; a pan-CAF marker) and (a) α-smooth muscle actin (α-SMA); (b) CD74, a transmembrane molecule involved in formation and transport of major histocompatibility (MCH) class II peptides; and (c) IL-6, an inflammatory cytokine. In PDAC xenografts from all 3 cell lines, α-SMA^+^ CAFs comprised the majority, with less contribution from CD74^+^ and IL-6^+^ CAFs. Silencing *HMGA1* or *FGF19* reduced the proportion of all 3 CAF subtypes (Figure 9, D and E, and Supplemental Figure 5, D and E). Together, these findings indicate that HMGA1 and FGF19 modulate CAF composition to induce the formation of a desmoplastic stroma in xenografted tumors.

### Hmga1 deficiency in KPC mice impairs tumor and stroma formation.

To investigate *Hmga1* in tumorigenesis, CAF composition, and stroma formation in mice with a competent immune system, we crossed KPC mice, in which PDAC develops more gradually (63), with mice with global deficiency of one or both *Hmga1* alleles (all on C57BL/6 backgrounds) and followed offspring for evidence of PDAC (abdominal distension, rectal prolapse, palpable tumors) or ill appearance (hunching, or decreased activity, oral intake, or weight; Table 1). Similar to prior reports (63), KPC mice (24 of 24 evaluable mice) developed pancreatic tumors by 14.1 weeks [median survival time]) (Supplemental Figure 6A). Tissue autolysis precluded further analyses in 2 mice that died at 8 and 16 weeks. A subset of KPC mice developed rectal prolapse (5 of 24) and/or ascites (3 of 24) (Table 1). In all cases, invasive pancreatic tumors developed (24 of 24) with pathology consistent with PDAC in most (92%; 22 of 24); 2 developed an undifferentiated sarcomatoid pancreatic tumor. By contrast, KPC mice with *Hmga1* heterozygosity had delayed tumorigenesis and prolonged survival (*n* = 9; median survival 17.0 weeks). One KPC/*Hmga1* heterozygous mouse developed a large salivary gland tumor at 7.4 weeks of age; the pancreas showed only rare foci of acinar ductal metaplasia. Of note, *Hmga1* heterozygous mice have normal life expectancy with no evidence of abnormal growth or development (48, 49). We also generated 1 KPC mouse null for *Hmga1*, which had a normal pancreas size and only rare foci of acinar ductal metaplasia at 22 weeks; it was sacrificed prior to any evidence of illness. *Hmga1*-knockout mice have decreased embryonic viability, whereas those that survive development are slightly small but appear grossly normal up to 30 weeks of age when they develop signs of premature aging (graying, osteopenia, decreased gait velocity) (49). We used ultrasound to confirm the presence of pancreatic tumors in a subset of mice (Supplemental Figure 6B).

To ascertain whether *Hmga1* deficiency alters pancreatic stroma development and fibrosis in KPC mice, we validated HMGA1 deficiency (via IHC), which showed robust HMGA1 intranuclear staining in KPC mice, less staining in KPC mice with *Hmga1* heterozygosity, and complete absence of HMGA1 in KPC/*Hmga1*-knockout mice (Supplemental Figure 6C). FGF19 staining paralleled results observed with HMGA1, with robust FGF15 staining in KPC mice, moderate staining in KPC/*Hmga1* heterozygous mice, and low levels in the KPC pancreas with *Hmga1* knockout (Supplemental Figure 6C). Similarly, fibrosis scores decreased in KPC mice with a deficiency of one *Hmga1* allele (Supplemental Figure 6, C and D, and Table 1), while the *Hmga1*-knockout mouse did not develop PDAC or fibrosis by 22 weeks. These data demonstrate that HMGA1 is required for pancreatic tumorigenesis and stromal formation in KPC mice.

### Hmga1 haploinsufficiency within pancreatic ductal epithelium is sufficient to mitigate tumor and stroma formation in KPC mice.

**Figure 10:**
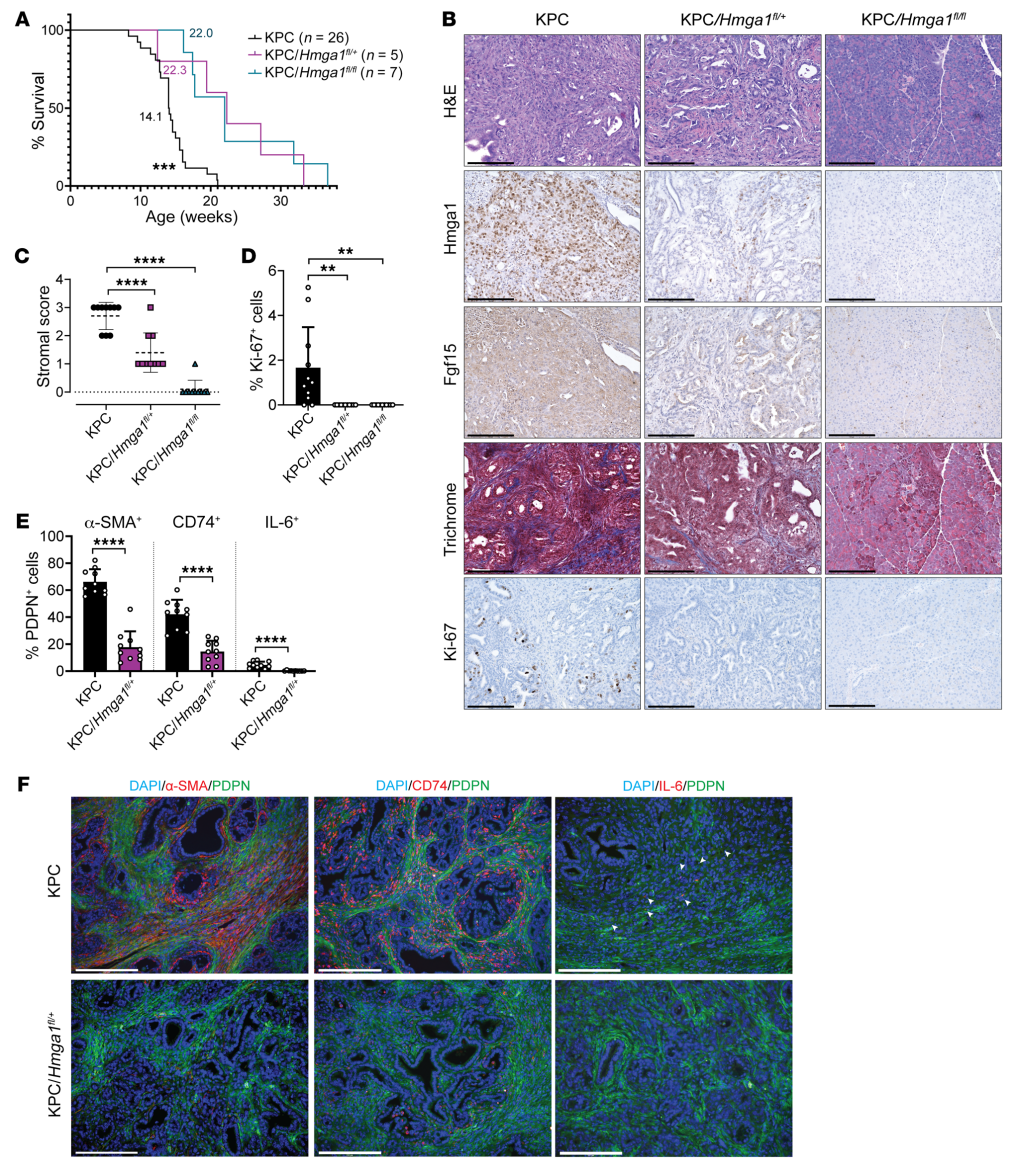
*Hmga1* haploinsufficiency within the pancreatic ductal epithelium is sufficient to mitigate tumorigenesis and fibrotic stroma formation in KPC mice. (**A**) Kaplan-Meier plot showing survival in KPC mice (*n* = 26, 11 males) compared to KPC with pancreatic ductal epithelial heterozygous *Hmga1* deficiency, KPC/*Hmga1^fl/+^* (*n* = 5, 3 males), or KPC mice with pancreas-specific homozygous *Hmga1* deficiency KPC/*Hmga^fl/fl^* (*n* = 7, 5 males). Median survivals are indicated. (**B**) Representative images showing H&E (top row), HMGA1 (second row), FGF15 (third row), fibrosis (trichrome; fourth row), and Ki-67 (bottom row). Scale bars: 200 μm. (**C**) Comparison of stroma fibrosis scores in KPC models. (**D**) Comparison of Ki-67–positive cells in KPC models with or without pancreas-specific *Hmga1* deficiency. (**E**) CAF composition and (**F**) representative IF images in KPC models with or without pancreas-specific *Hmga1* deficiency. Total CAF number ascertained by costaining with DAPI and for PDPN; α-SMA, CD74, and IL-6 were used to identity percentages of total CAFs positive for each marker. In **B**–**F**, data were based on 5 fields at ×20 magnification of tumors from 2 mice/genotype, *n* = 10 per condition. Data presented as mean ± SD from independent mice. **P < 0.01, ***P < 0.001, ****P < 0.0001 by log-rank (Mantel-Cox) test (**A**), 1-way ANOVA with Dunnett’s multiple-comparison test (**C** and **D**), or 2-tailed Student’s *t* test for α-SMA^+^ and CD74^+^ CAFs (data normally distributed) and Mann-Whitney test for IL-6^+^ CAFs (data not normally distributed) (**E**). Scale bars: 200 μm.

To determine whether *Hmga1* deficiency within the pancreatic ductal epithelium is sufficient to mitigate tumorigenesis and stroma formation, we generated KPC mice crossed with mice with one or both *Hmga1* alleles floxed, resulting in deletion of floxed alleles within pancreatic epithelium, including KPC mice with pancreas-specific heterozygous (KPC/*Hmga1^fl/+^*) or homozygous (KPC/*Hmga1^fl/fl^*) deletions. Surprisingly, loss of just a single *Hmga1* allele within the pancreas (KPC/*Hmga1^fl/+^*) was sufficient to delay tumorigenesis and prolong survival (*n* = 5; 22.3 weeks) in KPC mice, and survival was prolonged even more than what we observed for KPC mice with global *Hmga1* heterozygous deficiency (Figure 10A). Survival was also prolonged in mice with pancreas-specific deletion of both *Hmga1* alleles (*n* = 7; KPC/*Hmga1^fl/fl^*; 22.0 weeks) similar to the KPC/*Hmga1^fl/+^*, suggesting that loss of just a single *Hmga1* allele is sufficient to mitigate tumorigenesis in KPC mice. Accordingly, HMGA1 IHC in KPC/*Hmga1^fl/+^* or KPC/*Hmga1^fl/fl^* mice showed a decrease or absence of HMGA1 in tumors cells, respectively, and FGF15 staining also decreased in parallel with HMGA1 (Figure 10B). Moreover, fibrosis scores and Ki-67 decreased in KPC mice with *Hmga1* deficiency within pancreatic epithelium (Figure 10, B–D). Further, all 3 major CAF subtypes (by IF) decreased with pancreatic epithelial *Hmga1* deficiency (Figure 10, E and F). Together, these striking results demonstrate that the loss of just a single *Hmga1* allele within the pancreatic ductal epithelium is sufficient to mitigate tumorigenesis, stroma formation, and modulate CAF composition, thereby prolonging survival in KPC mice.

### HMGA1 and FGF19 are upregulated in human PDAC with exceptionally poor outcomes.

**Figure 11:**
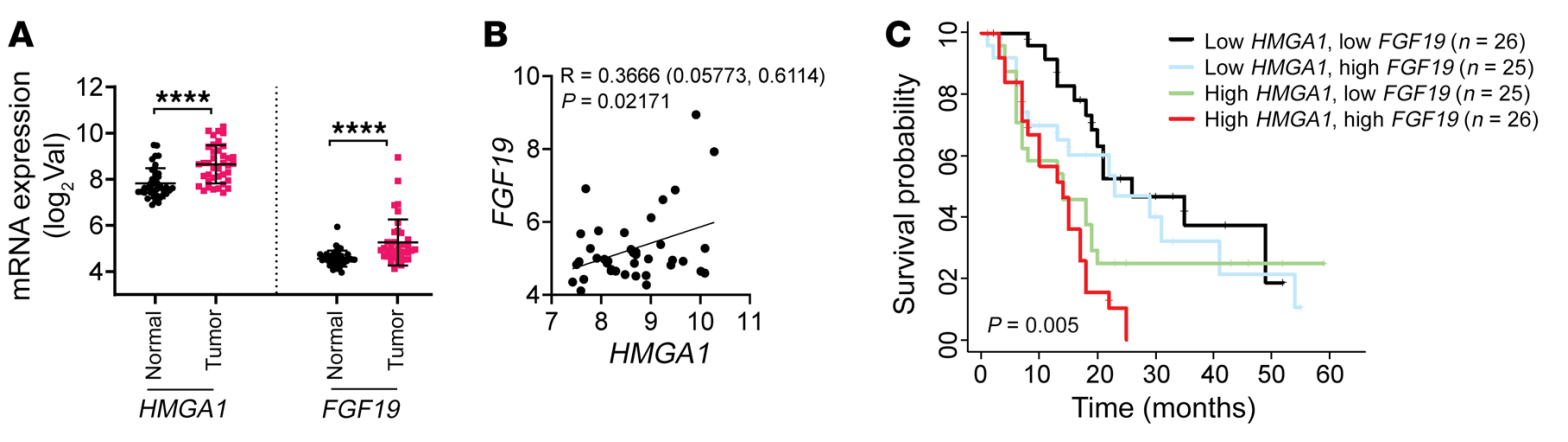
Overexpression of both *HMGA1* and *FGF19* in human PDAC defines a molecular subclass with extremely poor outcomes. (**A**) *HMGA1* and *FGF19* mRNA levels in paired nonmalignant tissue (labeled normal) and primary PDAC tumors (GSE15471); *n* = 36 for PDAC tumors and *n* = 36 for nonmalignant tissue. (**B**) *HMGA1* and *FGF19* expression is positively correlated in PDAC tumors (GSE15471; *n* = 36). (**C**) Kaplan-Meier plot showing poor overall survival of PDAC patients with both high *HMGA1* and *FGF19* expression (red, *n* = 26), high *HMGA1* and low *FGF19* expression (green, *n* = 25), low *HMGA1* and high *FGF19* expression (blue, *n* = 25), and low *HMGA1* and *FGF19* expression (black, *n* = 26) from GSE21501. Data presented as mean ± SD. Significance was evaluated by 2-tailed Student’s *t* test (**A**), Pearson’s analysis (**B**), or log-rank (Mantel-Cox) test (**C**). ****P < 0.0001.

To determine whether *HMGA1* and *FGF19* are relevant in human PDAC, we queried published data sets (GSE15471; *n* = 36 nonmalignant tissue, *n* = 36 tumor samples) (82). As expected, *HMGA1* was robustly upregulated in most human PDACs, consistent with prior studies (Figure 11A) (36, 38). By contrast, *FGF19* was variable, with tumors demonstrating low, moderate, or high expression (Figure 11A). However, *HMGA1* and *FGF19* correlated positively in all tumors, albeit weakly (Figure 11B). In another independent data set (GSE16515) (83), we validated similar patterns with consistent *HMGA1* overexpression and a broader range of *FGF19* expression (Supplemental Figure 7D). Since *HMGA1* is overexpressed in most tumors, whereas *FGF19* is upregulated in only a subset (~25%), we determined whether high expression of both *HMGA1* and *FGF19* predicts outcomes. In a PDAC database with survival data (GSE21501; *n* = 102 PDAC tumors) (84), we categorized PDAC tumors (*n* = 102) by quartiles based on relative expression of both genes, with the upper quartile representing tumors with highest expression of *HMGA1* and *FGF19* (red line; *n* = 26) and the lower quartile representing tumors with lowest expression of *HMGA1* and *FGF19* (black line; *n* = 26). We included a quartile with high *HMGA1* and low *FGF19* (green line; *n* = 25) and relatively low *HMGA1* with high *FGF19* (blue line; *n* = 25) (Figure 11C). Strikingly, tumors with high levels of both *HMGA1* and *FGF19* had worse overall survival (*P* = 0.005), indicating that this pathway is relevant to human PDAC and further underscoring FGF19 as a plausible therapeutic target for this highly recalcitrant molecular subtype (Figure 11C).

### BLU9931 decreases tumorigenesis and stroma formation in orthotopic PDAC models.

**Figure 12:**
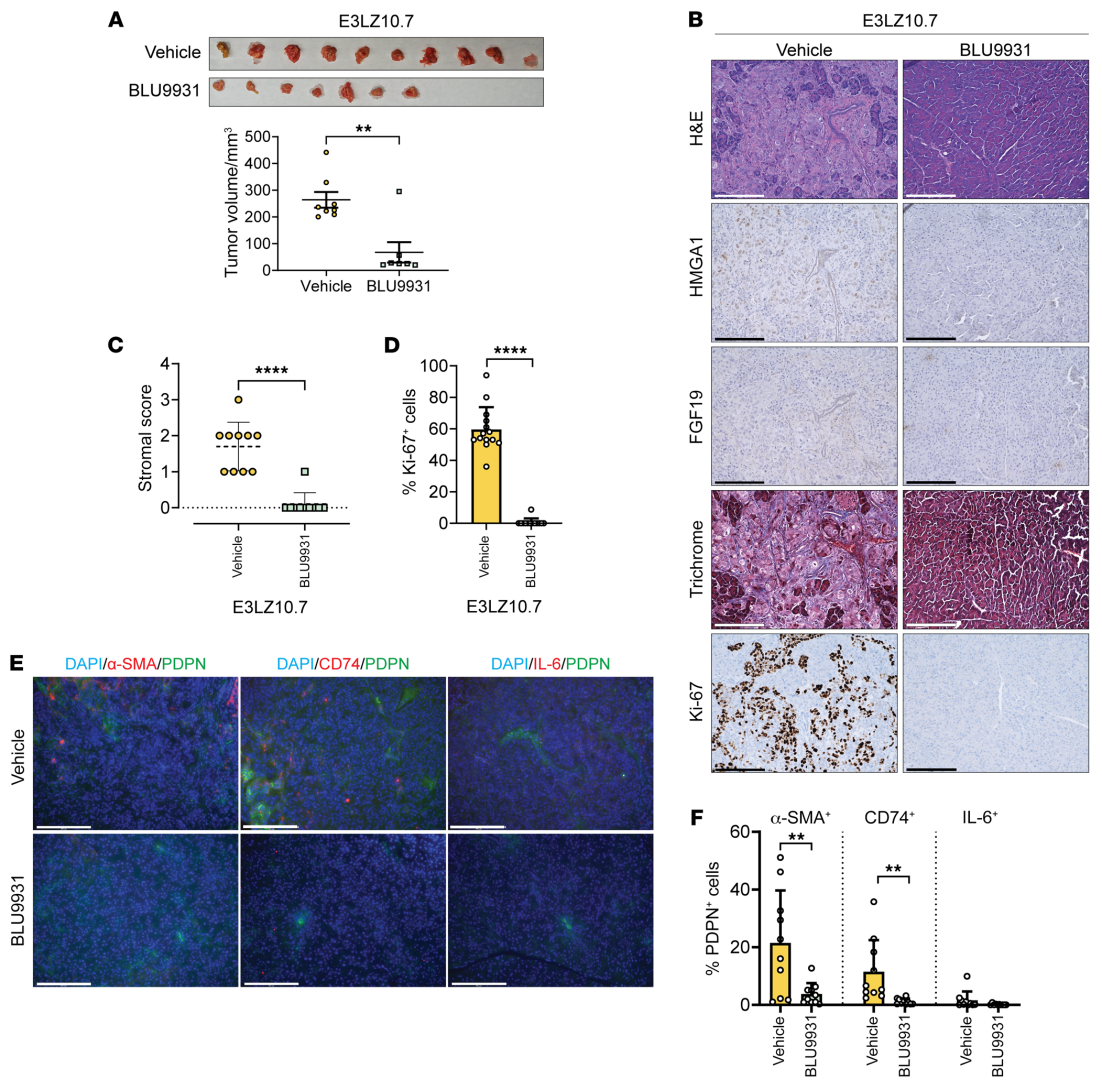
FGFR4 inhibition with BLU9931 decreases tumorigenesis and stroma formation in human PDAC orthotopic implants. (**A**) Tumors (top) and volume comparisons (bottom) from orthotopic implantation of E3LZ10.7 cells in mice treated with BLU9931 or vehicle control. Data presented as mean ± SEM. (**B**) Representative images (*n* = 10 per condition) of tumors stained with H&E (top row) and for HMGA1 (second row), FGF19 (third row), fibrosis (trichrome; fourth row), and Ki-67 (bottom row) in E3LZ10.7 orthotopic implants of mice treated with BLU9931 or vehicle. (**C**) Comparison of stromal fibrosis scores in E3LZ10.7 orthotopic implants based on a 3-point system. (**D**) Comparison of Ki-67^+^ cells in E3LZ10.7 orthotopic implants of mice treated with BLU9931 or with vehicle control. (**E**) Representative IF images of CAFs in E3LZ10.7 orthotopic implants of mice treated with BLU9931 or with vehicle. (**F**) Comparison of CAFs in E3LZ10.7 orthotopic implants of mice treated with BLU9931 or vehicle. Total CAF number ascertained by costaining with DAPI and for PDPN; α-SMA, CD74, and IL-6 were used to identify percentage of total CAFs positive for each marker. Data in **C**–**D** were based on 10 fields from 3 different mice/group at x20 magnification (*n* = 10/condition); data in **E** were based on 10 fields from 1 mouse/group at x20 magnification (*n* = 10/condition). Data presented as mean ± SD (**C**, **D**, and **F**). Significance was evaluated by Mann-Whitney test (**A**, **C**, and **D**) or 2-tailed Student’s *t* test for α-SMA^+^ and CD74^+^ CAFs (data normally distributed) and Mann-Whitney for IL-6^+^ CAFs (data not normally distributed) (**F**). **P < 0.01, ****P < 0.0001. Scale bars: 200 μm.

Because our primary goal is to identify actionable mechanisms in PDAC, we determined whether targeting the HMGA1/FGF19 pathway with BLU9931 mitigates tumor and stroma formation. We tested BLU9931 at doses established to reach pharmacologic levels in mice (64) in human PDAC xenografts from E2LZ10.7 cells (1 × 10^6^) injected into the midpancreas of immunosuppressed mice (NOD Scid γ, NSG). Once tumors reached a volume of 100–200 mm^3^ by ultrasound, mice were given BLU9931 twice daily by oral gavage (300 mg/kg or vehicle control) approximately 1 week following implantation. Mice underwent necropsy after 4 weeks of therapy when controls began to appear ill. Strikingly, there was a marked decrease in tumor volumes in mice treated with BLU9931, along with decreased staining for HMGA1, FGF19, Ki-67, and fibrosis (trichrome) (Figure 12, A–D). The 3 CAF subtypes also decreased with BLU9931 (Figure 12, E and F), suggesting that targeting FGFR4 with BLU9931 is a promising approach for human PDAC overexpressing *HMGA1* and *FGF19*.

**Figure 13:**
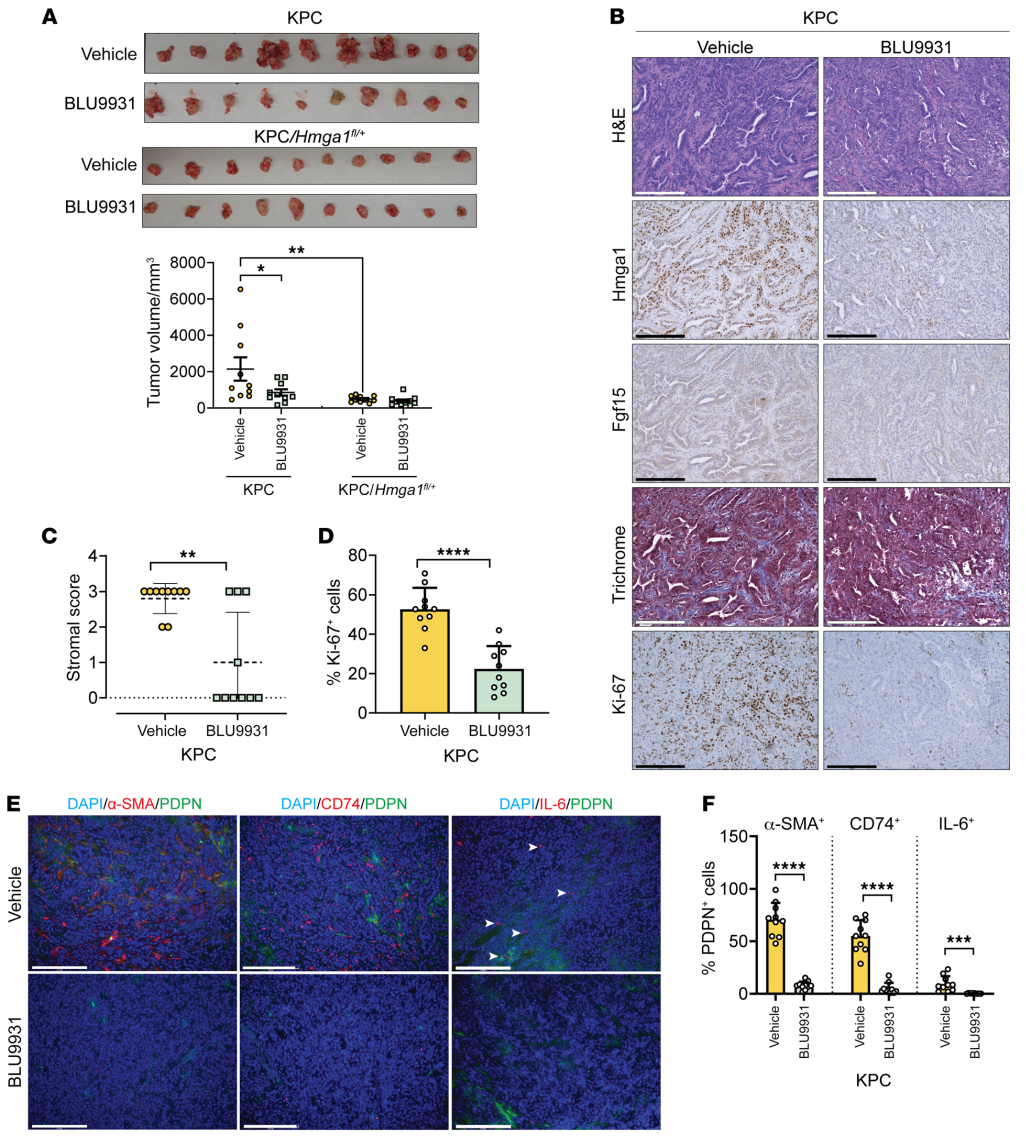
BLU9931 mitigates tumorigenesis and stroma formation in orthotopic implants from KPC PDAC cells. (**A**) Tumors (top) and volume comparisons (bottom) from orthotopic implantation of KPC xenografts mice treated with BLU9931 or vehicle control. Data presented as mean ± SEM. (**B**) Representative images (*n* = 10 per condition) of tumors stained with H&E (top row) and for HMGA1 (second row), FGF15 (third row), fibrosis (trichrome, fourth row), and Ki-67 (fifth row) in KPC orthotopic implants of mice treated with BLU9931 or vehicle control. (**C**) Stromal fibrosis scores shown as violin plots in KPC orthotopic implants based on a 3-point system. (**D**) Comparison of Ki-67^+^ cells in KPC orthotopic implants of mice treated with BLU9931 or vehicle control. (**E**) Representative IF images of CAFs. (**F**) Comparison of CAFs in KPC orthotopic implants of mice treated with BLU9931 or vehicle control. Total CAF number ascertained by costaining with DAPI and for PDPN; α-SMA, CD74, and IL-6 were used to identify different subtypes of CAFs positive for each marker. Data in **C**–**D** were based on 10 fields from 3 different mice/group at x20 magnification (*n* = 10/condition); data in **E** were based on 10 fields from 1 mouse/group at x20 magnification (*n* = 10/condition). Data presented as mean ± SD (**C**, **D**, and **F**). Significance was evaluated by Significance was evaluated by 1-way ANOVA with Tukey’s multiple-comparison test (**A**), Mann-Whitney test (**C**), 2-tailed Student’s *t* test (**D**), or 2-tailed Student’s *t* test for α-SMA^+^ and CD74^+^ CAFs (data normally distributed) and Mann-Whitney test for IL-6^+^ CAFs (data not normally distributed) (**F**). *P < 0.05, **P < 0.01, ***P < 0.001, ****P < 0.0001.

Next, we tested BLU9931 in syngeneic mice with an intact immune system and KPC orthotopic implants. After generating subcutaneous xenografts from KPC and KPC/*Hmga1^fl/+^* heterozygous cell lines with tumor volumes of approximately 100–200 mm^3^, tumor fragments were implanted surgically into the pancreas of mice. One week after implantation, we confirmed tumor formation (volumes of 100–200 mm^3^) by ultrasound, after which mice were divided into treatment arms with similar tumor volume distributions (*n* = 8–10/group): (a) KPC implants, BLU9931 treatment (twice daily oral gavage); b) KPC implants, vehicle control (twice daily oral gavage); c) KPC-*Hmga1* heterozygous implants, BLU9931 treatment; and (d) KPC-*Hmga1* heterozygous implants, vehicle control. Mice were followed by weekly ultrasounds and necropsies performed when recipients of KPC implants treated with vehicle control appeared ill (after 4 weeks). We discovered a marked decrease in tumor volume in recipients of KPC implants treated with BLU9931 compared with vehicle control (Figure 13A). Further, KPC implant recipients treated with BLU9931 had decreased levels of HMGA1, FGF19, fibrosis, and Ki-67 (Figure 13, B–D). Similar to KPC mice with *Hmga1* deficiency, the frequency of all CAF subtypes decreased (Figure 13, E and F). Recipients of KPC implants with *Hmga1* heterozygous deficiency had slightly smaller tumors than KPC mice treated with BLU9931. Although BLU9931 resulted in slightly lower mean tumor volumes in KPC/*Hmga1* heterozygous implants in addition to decreased HMGA1, FGF15, and Ki-67 staining, and 2 of 3 CAF subtypes, the changes were modest, as tumor growth was markedly diminished by *Hmga1* haploinsufficiency alone (Supplemental Figure 8, A–E). Taken together, these results indicate that HMGA1 drives PDAC tumor initiation, progression, and stroma formation, at least in part, by inducing *FGF19* expression and secretion. Moreover, this pathway can be disrupted with an FGFR4 inhibitor, BLU9931. Most importantly, overexpression of *HMGA1* and *FGF19* defines a subset of human PDAC with exceptionally poor outcomes, underscoring the need for further studies to assess targeting FGF19 in PDAC.

## Discussion

Alterations in chromatin regulators frequently occur in cancer, although most epigenetic modulators have eluded therapeutic targeting (85–87). For example, genes encoding chromatin regulators involved in pluripotency, *OCT4*, SOX2, *KLF4*, *NANOG*, and *LIN28*, are rarely mutated, but frequently overexpressed in cancer, thus rendering pharmacologic interventions challenging (87). Such factors are believed to reprogram the epigenome to a more plastic, stem-like state, thereby endowing tumor cells with the capacity to proliferate in a deregulated fashion, circumvent differentiation cues, evade therapy, and metastasize. HMGA1 chromatin regulators are oncofetal proteins that enhance cellular reprogramming by upregulating pluripotency networks (47, 88, 89). Similar to pluripotency factors, *HMGA1* is rarely mutated, but almost universally overexpressed in aggressive cancers, consistent with a fundamental role in tumorigenesis (47, 88). Indeed, HMGA1 is among the most abundant, nonhistone chromatin-binding proteins within nuclei of cancer cells where it induces genes expressed in stem cells and tumor progression (30, 38, 46, 48, 88, 89).

While many studies show *HMGA1* upregulation in PDAC (36, 38, 50, 61), transcriptional networks governed by HMGA1 that could be targeted in therapy remained elusive until now. We identified a single growth factor, FGF19, that fosters not only oncogenic properties, but also signals within the microenvironment to induce fibrotic desmoplasia. This mechanism is potentially unique because it involves both tumor cell–intrinsic and microenvironmental interactions that collaborate during tumor progression. Intriguingly, we recently found that HMGA1 causes bone marrow fibrosis during progression in mouse models of chronic myeloid malignancies (*JAK2^V617F^* myeloproliferative neoplasms), suggesting that fibrosis mediated by HMGA1 is relevant to diverse tumors (49). Importantly, HMGA1 also regulates transcriptional networks involved in cell cycle progression (E2F targets, G_2_/M checkpoint, mitotic spindle) in myeloid malignancies, although *FGF19* and bile acid metabolic genes are unique to PDAC cells. Surprisingly, silencing *FGF19* recapitulates most, but not all, phenotypes associated with *HMGA1* silencing, suggesting that it is an important transcriptional target, although other HMGA1 transcriptional networks clearly contribute to PDAC carcinogenesis in our models.

Prior studies revealed mutations and epigenetic alterations that arise early in pancreatic carcinogenesis, although this has not impacted therapies (3). Less is known about later mechanisms driving progression. Clonal evolution studies suggest that PDACs evolve over many years, or even decades, which could foster clonal diversity and facilitate tumor progression (90). Another vexing characteristic of PDAC is the desmoplastic stroma composed of fibrotic scar tissue and CAFs, which also exhibit heterogeneity (9, 10). Although studies of CAF signaling and biophysical properties of stroma suggest that desmoplasia fuels tumor progression, the stroma restrains tumor growth in KPC models (7, 8). These studies, together with our results, suggest that the stroma has multiple functions, which may depend on tumor stage and properties of tumor cells, and stromal composition. The stroma could provide an initial barrier that is circumvented as tumor cells become more plastic (18). While we could not dissect the contribution of the stroma in isolation, our models suggest that HMGA1 and FGF19 collaborate to promote tumor progression and stroma formation. Because HMGA1 proteins are detectable only in late-stage precursor lesions (pancreatic intraepithelial neoplasia [PanIN] 3) or invasive tumors, this mechanism may be relevant later in carcinogenesis when tumor cells invade and metastasize (38). Of note, we found lower frequencies of IL-6^+^ CAFs in KPC mice compared with other studies (19). Although the reason for this is unknown, inflammatory signals may vary in different mouse colonies from factors such as the microbiome. Despite these differences, however, tumors formed within a time frame similar to those of published studies with KPC mice. Together, our work reveals a therapeutic target relevant to a newly defined molecular subclass of human PDAC characterized by high expression of *HMGA1* and *FGF19*. Indeed, gene expression and survival data indicate that such tumors are among the most rapidly lethal PDACs.

FGF19 is a pleiotropic, hormone-like protein that regulates lipid, carbohydrate, and bile acid metabolism through the receptor FGFR4 (72). Released from the ilium into enterohepatic circulation after exposure to bile salts in postprandial states, FGF19 dampens further bile acid release (72). *FGF19* is also expressed in embryonic stem cells (91). In mice, FGF15 is required for embryogenesis and liver regeneration (92), and FGF15 induces hepatocellular carcinoma (HCC) when overexpressed in skeletal muscle, presumably through paracrine effects (71). *FGF19* is also overexpressed in human HCC harboring amplifications involving the FGF19 locus (chromosome 11q13) (93), which led to the development of clinical inhibitors (64, 75–77). A recent study in HCC, however, showed only modest responses to an FGFR4 inhibitor (75), although chemically induced HCC in mice with *Fgf15* deficiency show less fibrosis (74), suggesting that FGF15 fosters fibrosis in HCC. *HMGA1* is also upregulated in human HCC (94, 95), and *FGF19* is overexpressed or amplified in other tumors with *HMGA1* overexpression (77). In a PDAC cell line, GLI/ERK signaling upregulates *FGF19* and xenograft tumorigenesis (96), and our GSEA analyses link HMGA1 to ERK networks (Supplemental Table 1), consistent with HMGA1 as a central hub through which multiple oncogenic pathways converge. In PDAC models, *FGF19* promotes tumor growth and stroma formation. Moreover, KPC mice with loss of a single *Hmga1* allele within pancreatic ductal epithelium exhibit increased tumor latency, less fibrosis, and decreased FGF15 immunoreactivity, further supporting a collaborative role for HMGA1 and FGF15 in tumorigenesis and fibrotic desmoplasia (Figure 10, B and C).

In human PDAC, *FGF19* expression is more variable than *HMGA1*, the latter of which is upregulated in most tumors (36, 38). Why *FGF19* is induced in only a fraction of tumors remains unclear. Pancreatic carcinogenesis may proceed through stepwise accumulation of mutations, or chromothripsis, whereby thousands of clustered chromosomal rearrangements occur simultaneously (3, 97). The complex genome likely contributes to PDAC heterogeneity, and some genetic alterations may affect *FGF19* expression. Notably, FGF19 only partially restores proliferation in cells with *HMGA1* silencing, indicating that other HMGA1 pathways foster tumorigenesis. Our transcriptomes reveal multiple HMGA1 pathways and further investigation could reveal other actionable mechanisms. However, *FGF19* deficiency recapitulates most effects of *HMGA1* silencing and our KPC studies are consistent with FGF15 as a downstream HMGA1 effector. Despite the circumscribed population of human tumors with both *HMGA1* and *FGF19* overexpression, these data delineate a molecular subclass with worse outcomes that could be targeted in therapy (98).

KRAS-driven tumors, and PDAC in particular, have proven formidable therapeutic challenges. Therapies that target KRAS are emerging, although their efficacy in PDAC is unknown (99, 100). While inhibitors of chromatin regulators, such as bromodomain proteins, show efficacy, successes in PDAC are lacking (101). Growth factors provide attractive targets because they can be neutralized by antibodies or receptor blockade. Our work illuminates HMGA1 and FGF19 as key players in PDAC tumorigenesis and stroma formation. Most importantly, this pathway is conserved in a subset of human tumors with exceptionally poor outcomes. Together, we discovered what we believe is a previously undescribed paradigm whereby tumor cells collaborate via HMGA1 and FGF19 to drive progression, thus illuminating FGF19 as a rational therapeutic target for a molecular subclass composed of the most aggressive human PDACs. 

## Methods

Detailed methods, statistical analyses, and reagents are provided in the supplemental material, including culture medium, primers, and antibodies (Supplemental Tables 2–4). RNA sequencing data were deposited into the NBCI Gene Expression Omnibus (GEO GSE222890). See complete unedited blots in the supplemental material.

## Author contributions

LR conceptualized the project. LC and BW drafted parts of the manuscript, and LR wrote the final draft, which was reviewed by all authors prior to submission. LC, BW, JHK, LZL, SS, IH, SYC, LL, LX, TH, MH, WJS, SI, GG, LMC, KG, LW, and LR performed experiments and analyzed data. EJ, LZ, KR, KG, and LMC provided reagents and guidance with experiments. LW, BW, LC, LZL, and WJS interpreted histology.

## Supplementary Material

Supplemental data

Supplemental table 1

## Figures and Tables

**Table 1:**
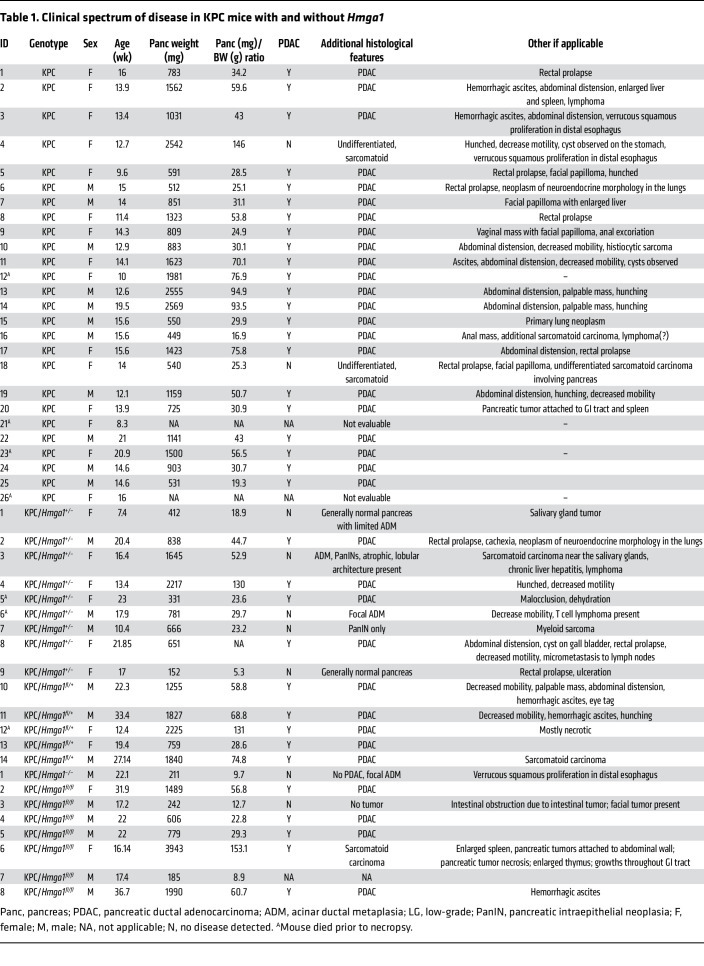
Clinical spectrum of disease in KPC mice with and without *Hmga1*

## Version 1 03/15/2023

Electronic publication
