# Supplementary material for: HMGA1 induces FGF19 to drive pancreatic carcinogenesis and stroma formation
Source: J Clin Invest. 2023 Mar 15;133(6):e151601. doi: 10.1172/JCI151601 (PMC10014113; doi:10.1172/JCI151601)
Supplement: Supplemental data [file jci-133-151601-s116.pdf]

Materials and Methods

Cell lines: E3LZ10.7 (1), MIA PaCa-2, and AsPC-1 (American Type Culture Collection, ATCC) were cultured as recommended (**Supplemental Table 2**) with regular STR validation and mycoplasma testing.

Genetic mouse models: Xenograft tumors were generated by implanting PDAC cell lines (10^5, or limiting dilutions of $5x10^4$, 10^4) in 10-week-old female nude mice (Taconic). KPC (*LSL-Kras^{G12D}*; *LSL-Trp53^{R172H} Pdx*-Cre-1) (2) models were crossed to mice with global deficiency of one or both *Hmga1* alleles (C57Bl6 backgrounds) (3, 4). To generate pancreas-specific deficiency of *Hmga1*, we crossed KPC mice to *Hmga1$^{fl/fl}$* (3, 4) onto C57Bl6 backgrounds (>5 generations) to generate KPC/*Hmga1$^{fl/fl}$* and KPC/*Hmga1$^{fl/+}$*. Both sexes were used (specified in Table 1), and mice were sacrificed when they developed evidence of tumors (abdominal distension, rectal prolapse, palpable tumors) or otherwise appeared ill (hunching or decreased activity, oral intake, or weight). Tissues were fixed in formalin before processing for histology. Histopathology was evaluated by LW, WJS, LZL, and tissues from KPC crosses were read blindly by a pathologist with expertise in PDAC (LW). Mice were housed in sterile, pathogen-free environments with free access to food and water.

KPC cell lines and orthotopic implantation: KPC cell lines were derived from KPC mice with both *Hmga1* alleles intact or pancreas-specific KPC/*Hmga1$^{fl/+}$* as described (5) except for modifications in cell strainer (70 um), antibiotics, and antifungal agents (**Supplemental Table 2**). Genotype of all KPC cell lines was validated (Transnetyx, Inc); both KPC cell lines became biallelic for the *TP53* mutation in culture prior to implantation. Cells were implanted subcutaneously (10^6) until tumor volumes were >100-200 mm^3, after which tumor fragments (100-150 mm^3) were implanted orthotopically as described (5, 6). Genotype of all KPC cell lines was validated (Transnetyx, Inc); both KPC cell lines became biallelic for the *TP53* mutation in culture prior to implantation. Cells were implanted subcutaneously (10^6) until tumor volumes were >100-200 mm^3, after which tumor fragments (100-150 mm^3) were implanted orthotopically as described (5, 6). As a wild type control for *Hmga1* and *Fgf15* expression to compare to KPC cell lines, normal mouse pancreas (age 9 weeks; male) was snap-frozen in liquid nitrogen within 2 min of extraction, and ground on dry ice in Trizol for subsequent RNA extraction.

BLU9931: BLU9931, a gift from Blueprint Medicines Corporation (Cambridge, MA), was administered one week after orthotopic implants and ultrasound confirmation of tumors. Mice received BLU9931 (300 mg/kg) or vehicle

control (0.5% carboxymethylcellulose) twice daily by oral gavage. Weekly ultrasounds were performed to monitor tumor growth.

Lentivirus mediated shRNA silencing in cells: Lentiviral delivery of plasmids encoding shRNA targeting *HMGA1* (sh*HMGA1* 1: TRCN0000018949, sh*HMGA1* 2: TRCN0000018952), *FGF19* (sh*FGF19* 1: TRCN0000040259 and sh*FGF19* 2: TRCN0000040261) (RNAi Consortium/TRC) or control lentiviral (expressing empty vector control (SHC001, Millipore Sigma) were used for gene silencing as described (3, 4). Cells were transduced using polybrene (8 μg/ml, Millipore Sigma) for 24 h at 37°C and selected with puromycin (5.3 μg/ml E3LZ10.7 cells; 1 μg/ml MIA PaCa-2 cells; 2 μg/ml AsPC-1 cells) for seven days.

Proliferation: Proliferation was assessed using the 3-(4,5-dimethylthiazol-2-yl)-5-(3- carboxymethoxyphenyl)-2-(4-sulfophenyl)-2H-tetrazolium (MTS; Promega) and phenazine methyl sulfate (PMS; Promega) according to manufacturer's instructions. To test the effects of rFGF19, cells were rendered quiescent by serum deprivation (0.25% FBS) for 16-24h and incubated with 100ng or 300ng (depending on cell line as delineated) of recombinant human FGF19 (R&D, 969-FG-025/CF).

Migration/invasion assays: Migration and invasion were assessed using transwell inserts (8 μm pores). Transwell inserts were coated with a thin layer of Matrigel for invasion (Corning). Before seeding into upper chambers, cells (~$1.5x10^5$ cells) were maintained in serum-free medium for 24 h and treated with 10 μM of Cytosine β-D-arabinofuranoside (AraC, Sigma) to block proliferation (7). Complete medium was added to the lower chambers as a chemoattractant; migration or invasion was assessed after 24 h by fixation (4% paraformaldehyde) and crystal violet stain (0.2%; Sigma) for enumeration.

Clonogenicity: Cells ($5x10^3$) were resuspended in complete medium containing agarose (0.4%; ThermoFisher Scientific) and placed onto supporting layers of complete medium and agarose (0.8%). Selection medium was added every 3-4 days, and colonies (diameter ≥50 μm) were stained with crystal violet (0.01%; Sigma) and counted after three weeks.

Three-dimensional (3D) sphere-formation: 3D sphere-formation was performed as described with the following modifications (8). Cells ($2X10^3$) were cultured in 24 well low attachment plates (Corning) with DMEM:F12 (Gibco)

media supplemented with B27 (2%; Gibco), N2 (1% Gibco), and recombinant, human bFGF and EGF (both at 20 ng/ml; Thermo Fisher Scientific) for 14 days after which spheres (diameter $\geq$50 μm) were counted.

RNA sequencing (RNAseq), gene set enrichment analysis (GSEA): After assessing quality (RNA 6000 nano total RNA kit; 2100 Bioanalyzer; Agilent), samples with RNA integrity numbers (RIN) >9 underwent rRNA depletion and enrichment for poly-A (NEBNext Poly(A) mRNA Magnetic Isolation Module). RNA was converted to indexed, strand-specific RNA sequencing libraries (TruSeq stranded mRNA kit, Illumina) following the manufacturer's recommendation. RNAseq libraries were pooled and sequenced (HiSeq 2000, Illumina) to a read depth of ~30,000,000 with single-end 97 base pair reads/sample. Raw reads were 'cleaned' using 'Fastq quality trimmer by sliding window' and aligned to the human reference genome (hg38) with TopHat using strand-specific parameters (9). Transcript abundance (fragments per kilobase/million reads, FPKM) and differential expression estimates were generated (Cuffdiff2.1.1) using RefSeq followed by GSEA ; MSigDb) for Hallmark and curated genes (10, 11). Data were deposited into the Gene Expression Omnibus (GSE222890).

Gene expression: We queried transcript abundance for *HMGA1* and *FGF19* from human RNAseq datasets (GSE15471, GSE16515) (12, 13). Boxplots, scatterplots, and Pearson correlations were calculated using R statistical software suite and standard functions as reported (4).

RNA isolation and quantitative reverse transcriptase (RT) PCR analysis (qPCR): Total RNA from cells was isolated using Trizol reagent (Invitrogen) and the Direct-zol RNA miniprep kit (Zymo Research) according to the manufacturer's protocol. RNA (500 ng) was used for generating cDNA using the High-capacity cDNA Reverse Transcription kit (Applied Biosystems) according to the manufacturer's protocol. Expression for genes of interest with primers (**Supplemental Table 3**) was detected in human and mouse samples by quantitative PCR (qPCR), and reactions were performed using Power SYBR Green Master Mix (Applied Biosystems) on QuantStudio 3 (Applied Biosystems). Relative expression was determined as described with *HuPO* as a loading control in human cells and *Gapdh* as a loading control in mouse samples (14).

Chromatin Immunoprecipitation: Cells (10^7) were washed with phosphate-buffered saline (PBS) and crosslinked with formaldehyde (1%) for 10 minutes at room temperature. Glycine (1.25 M) was added to quench the cross-linking reaction, and cells were subsequently washed twice with cold PBS. Cells were lysed in cell lysis buffer (20 mM Tris pH 8.0, 85 mM KCl, and 0.5% NP40) containing protease inhibitors (Millipore Sigma) and centrifuged

at 430 *g* for 5 minutes at 4°C to separate cytoplasmic and nuclear fractions. Nuclear pellets were collected, lysed in nuclei lysis buffer (10 mM Tris-HCl pH 7.5, 1% NP40, 0.5% Na Deoxycholate, 0.1% SDS) containing protease inhibitors, transferred into a 1 ml milliTUBE (Covaris) and sheared using Covaris S220 (Covaris) sonicator. An aliquot of sheared chromatin (10%) was prepared as input. The sheared chromatin was immunoprecipitated by incubating with antibodies and Protein A/G Dynabeads (mixed 1:1, ThermoFisher Scientific) overnight while rotating at 4°C with the specified antibodies (**Supplemental Table 4**). Immunoprecipitates were washed with wash buffer (10 mM Tris-HCl pH 7.5, 1% NP40, 0.1% SDS) and eluted with ChIP elution buffer (50 mM $NaHCO_3$ and 1% SDS). Immunoprecipitated DNA samples were treated with RNase A (ThermoFisher Scientific) and proteinase K (ThermoFisher Scientific) and subsequently purified (QIAquick PCR Purification Kit; Qiagen). ChIP DNA was subjected to qPCR analysis using Power SYBR Green Master Mix (Applied Biosystems). MatInspector *in silico* transcription factor binding site prediction algorithm (15) was used to design primers for ChIP-qPCR analysis. (Primers are listed in **Supplemental Table 3**). ChIP-qPCR results are represented as a percentage of input based on mean quantity derived from a standard curve (4, 14).

Luciferase constructs and reporter assay: Luciferase reporter plasmids expressing *FGF19* promoter sequences containing predicted HMGA1 binding sites (-1144, -1046, -816, and -756 base pairs from the transcription start site) determined by MatInspector (15) were generated by PCR and cloned into pLightswitch_prom *Renella luciferase* plasmid (Switchgear Genomics). Sequences of the constructs were confirmed by Sanger sequencing. E3LZ10.7 cells with or without *HMGA1* silenced were transfected using Lipofectamine 3000 (Thermo Fisher Scientific) and a mixture of *Renella luciferase* vector (320 ng) and control PGK-*Firefly luciferase* vector (100 ng; Promega). At 48 h following transfection, cells were lysed using cell lysis buffer (Promega), and luciferase activity was measured. Renella luciferase activity was measured using the LightSwitch assay reagent (SwitchGear Genomics), while firefly luciferase activity was measured using the Luciferase assay reagent (Promega). Expression of the reporter gene was calculated as fold induction over the full-length promoter construct.

In vivo limiting dilution assay: Cells were injected into both flanks of the nude mice in a 1:1 mixture of PBS:Matrigel (Corning); AsPC-1 studies were performed without Matrigel due to supply chain disruptions during the pandemic. Mice were followed biweekly for tumor formation and underwent sacrifice and necropsy once tumor diameter reached 10 mm in mice injected with control cells. Tumor initiator frequency was calculated using

Extreme Limiting Dilution Analysis (16) where *P* value was determined by the chi-square test. Tumor volume was calculated as described (17) with volume = 0.5 x l x w^2 (l: length; w: width of tumor).

Immunoprecipitation (IP): Isolated protein (3 mg) was used for FGF19 IP in MIA PaCa-2 ± *HMGA1* or *FGF19* silencing. MIA PaCa-2 cell lysates were incubated overnight at 4°C with 40 μl of Protein A/G beads (ThermoFisher Scientific) coated with 1 μg of FGF19 antibody (Santa Cruz biotechnology, H-12/sc-390621) to immunoprecipitate FGF19. IPs were separated on a gradient bis-tris gel (4-12%), and FGF19 was detected with an antibody raised in rabbit (Cell Signal Technologies, D1N3R/#83348).

Immunohistochemistry and immunofluorescence: Hematoxylin & eosin (H&E) and immunohistochemistry (IHC) of xenograft sections and KPC pancreas were performed after formalin fixation and paraffin embedding as described previously (4, 18, 19). Slides were rehydrated in xylene and ethanol series after baking at 70°C for 1 h. Antigen retrieval was performed using preheated Target Retrieval Solution (Dako, S1699) for 20 minutes at 68ºC followed by 20 minutes at 99ºC. Slides were allowed to cool for 30 minutes followed by Tris Buffered Saline (TBS) with 0.5% Tween (Sigma Aldrich, TBST) washes. Slides were then blocked in 3% H_2O_2 for 30 minutes followed by Dako dual endogenous enzyme block (Dako, S200389-2) for 30 minutes and washed with TBS with 0.5% Tween and incubated with 3% Bovine Serum Albumin solution for 30 minutes. Tissues were incubated overnight at 4°C with primary antibodies (**Supplemental Table 4**) diluted in Dako antibody diluent (Dako, S080983-2). Tissues were subsequently washed in TBS with 0.05% Tween 20 and incubated with secondary antibodies (**Supplemental Table 4**) conjugated with fluorescence or horseradish peroxidase and visualized with diaminobenzidine (DAB+) solution (Dako, K346811-2). Tissues were mounted in VECTASHIELD® Antifade Mounting Medium with 4'-6-diamido-2-phenylindole (DAPI, Vector Laboratories, H-1200-10) for immunofluorescence or counter-stained with hematoxylin (Sigma Aldrich, MHS16) for 30 seconds, rinsed with tap water for 5 minutes, dehydrated in ethanol and xylene series, and mounted in adhesive (Fisherbrand™ Permount™ Adhesive; Fisher, SP15-100). Quantitative analysis of immunofluorescence (IF) and immunohistochemistry (IHC) staining was performed using QuPath (Version: 0.3.2). For quantitative comparisons of cancer-associated fibroblast (CAF) composition, we selected fields of 1000-2000 cells based on DAPI staining at 20x magnification and enumerated total 1154 ± 549 CAFs (mean ± SD CAFs/field) based on staining for podoplanin (PDPN, a "panCAF" marker). CAF composition was calculated as the percent of total PDPN+ cells that co-stain for another CAF marker, including and: 1) α-smooth muscle actin (α-SMA), 2) CD74,

a transmembrane molecule involved in the formation and transport of major histocompatibility (MCH) class II peptides, and, 3) IL6, an inflammatory cytokine. A total of 10 fields were selected in tumors from 1-2 mice for each condition.

Trichrome stain and analysis: As described previously, formalin-fixed, paraffin-embedded slides from xenografts and KPC tumors were prepared as described (18). Sections were stained with trichrome stain (Masson's, Abcam) according to the manufacturer's protocol. Fibrosis scores were assigned based on area staining with trichrome: 0 (<5%); 1 (5-30%); 2 (30-60%), 3 (>60%).

Immunoblotting: Total proteins were isolated from PDAC cells using RIPA buffer (Millipore Sigma) containing Halt protease and phosphatase inhibitor cocktail (ThermoFisher Scientific). Lysates (20 or 30 ug; with the same quantity loaded per lane per run) were separated on a gradient (4-12%) bis-tris gel (ThermoFisher Scientific) via SDS-polyacrylamide gel electrophoresis, transferred onto a polyvinylidene fluoride membrane (ThermoFisher Scientific), and analyzed using various primary antibodies (**Supplemental Table 4**). Beta-Actin (β-Actin) was used as a loading control in all Western blots. Most bands (HMGA1, β-Actin) on western blots were visualized with horse radish peroxidase (HRP) using a commercial imager (ChemiDoc XRS+; Bio-Rad) for the following: **Figures 1B, 3E** (AsPC-1), **3H**, **4B** (AsPC-1), **7C**, **7E**; **Supplemental Figures 1C**, **2C**, **3A**, **7C**. Because FGF19 bands were difficult to detect with HRP and the prior imager (ChemiDoc XRS+; Bio-Rad), we used a more sensitive, near infra-red fluorescence detection system (Odyssey CLx Imager; LI-COR Biosciences) to compare FGF19 and β-Actin in E3LZ10.7 and MIA PaCa-2 cells (**Figures 3E, 4B).** FGF19 (Santa Cruz), HMGA1 (Abcam), and β-Actin (Cell Signaling Technologies) were detected on the same blots shown in each figure section. HMGA1 and β-Actin were detected at 800 nm wavelength, and FGF19 was detected at 700 nm on the same blot in each figure section (Odyssey CLx Imager; LI-COR Biosciences). In blots from MIA PaCa-2 cells, the blot was stripped for 30 minutes at 37 ºC in stripping buffer (ThermoFisher Scientific). The blot was washed 3 times with TBS with 0.1% tween and re-probed with FGF19 antibody (Santa Cruz). FGF19 was then detected at 700 nm wavelength (Odyssey CLx Imager; Li-COR Biosciences).

Enzyme-linked immunoassay (ELISA) and *cytokine array*: For cytokine secretion analyses, cells were cultured in serum-free medium without phenol red for 24 h, after which medium was collected and centrifuged at 2,500 *g* for 10 minutes, and the resulting supernatant was concentrated onto filters (Amicon Ultra centrifugal filters – 10

k; Millipore Sigma). The concentration of secreted proteins was measured using a commercial protein assay (Coomassie Plus protein assay reagent; ThermoFisher Scientific). Total secreted proteins (200 ng for E3LZ10.7 and AsPC-1 cells, and 250 μg for MIA PaCa-2 cells) were used to quantitatively assess FGF19 by ELISA (RayBiotech) according to the manufacturer's protocol. Total secreted protein (750 μg) was used to quantify cytokines secreted in E3LZ10.7 cells using the Proteome Profiler Human XL Cytokine Array Kit (RnD Systems) according to manufacturers' protocol.

Flow cytometry: For the detection of total FGFR4 and phosphorylated FGFR4, cells (10^6) were fixed and permeabilized in solution (Cytofix/Cytoperm; BD Bioscience; 554714), washed twice in buffer (1x BD Perm/Wash buffer; BD Pharmingen), and incubated on ice for 30 minutes with rabbit anti-total FGFR4 (Abcam; ab5481) or rabbit anti-phosphorylated FGFR4 (Tyr754, ThermoFisher Scientific; PA5-64582) in the same buffer (1x BD Perm/Wash buffer; BD Pharmingen). Following two washes in the same buffer, cells were incubated with anti-rabbit IgG (Alexa Fluor Plus 680-labeled; ThermoFisher Scientific; A32734). Antibody-stained cell suspensions were acquired on a fluorescence-activated cell sorting machine (BD Biosciences FACS LSRII), and data were analyzed using commercial software (FlowJo software; Tree Star Inc).

Statistical analysis: When comparing 2 groups, statistical significance was determined using a two-tailed *student's t*-test when normally distributed (ascertained by Ryan-Joyner and D'Agostino-Pearson tests). If not normal, the Mann-Whitney test was used. When comparing more than 2 groups, statistical significance was determined using a one-way ANOVA with Dunnett's or Turkey's multiple comparisons (Prism 9, GraphPad Software). Survival analyses were performed under the assumption of Cox proportional hazards and evaluated by log-rank test. Tumor initiator cell frequency was compared by chi-square distributions. $P<0.05$ was considered significant.

Approvals: All mouse studies were approved by the JHU Institutional Animal Care and Use Committee (IACUC).

References

1. Feldmann G, et al. Blockade of hedgehog signaling inhibits pancreatic cancer invasion and metastases: a new paradigm for combination therapy in solid cancers. *Cancer Res.* 2007;67(5):2187–2196.

2. Hingorani SR, et al. Trp53R172H and KrasG12D cooperate to promote chromosomal instability and widely metastatic pancreatic ductal adenocarcinoma in mice. *Cancer Cell* 2005;7(5):469–483.

3. Gorbounov M, et al. High mobility group A1 (HMGA1) protein and gene expression correlate with ER-negativity and poor outcomes in breast cancer. *Breast Cancer Res. Treat.* 2020;179(1):25–35.

4. Li L, et al. HMGA1 chromatin regulators induce transcriptional networks involved in GATA2 and proliferation during MPN progression. *Blood* 2022;139(18):2797–2815.

5. Foley K, et al. Semaphorin 3D autocrine signaling mediates the metastatic role of annexin A2 in pancreatic cancer. *Sci. Signal.* 2015;8(388):ra77.

6. Fujiwara K, et al. Interrogating the immune-modulating roles of radiation therapy for a rational combination with immune-checkpoint inhibitors in treating pancreatic cancer. *J. Immunother. Cancer* 2020;8(2). doi:10.1136/jitc-2019-000351

7. Flamini MI, et al. Retinoic acid reduces migration of human breast cancer cells: role of retinoic acid receptor beta. *J. Cell Mol. Med.* 2014;18(6):1113–1123.

8. Zhao S, et al. CD44 expression level and isoform contributes to pancreatic cancer cell plasticity, invasiveness, and response to therapy. *Clin. Cancer Res.* 2016;22(22):5592–5604.

9. Trapnell C, Pachter L, Salzberg SL. TopHat: discovering splice junctions with RNA-Seq. *Bioinformatics* 2009;25(9):1105–1111.

10. Subramanian A, et al. Gene set enrichment analysis: a knowledge-based approach for interpreting genome-wide expression profiles. *Proc. Natl. Acad. Sci. USA* 2005;102(43):15545–15550.

11. Mootha VK, et al. PGC-1alpha-responsive genes involved in oxidative phosphorylation are coordinately downregulated in human diabetes. *Nat. Genet.* 2003;34(3):267–273.

12. Badea L, et al. Combined gene expression analysis of whole-tissue and microdissected pancreatic ductal adenocarcinoma identifies genes specifically overexpressed in tumor epithelia. *Hepatogastroenterology* 2008;55(88):2016–2027.

13. Pei H, et al. FKBP51 affects cancer cell response to chemotherapy by negatively regulating Akt. *Cancer Cell* 2009;16(3):259–266.

14. Xian L, et al. HMGA1 amplifies Wnt signalling and expands the intestinal stem cell compartment and Paneth cell niche. *Nat. Commun.* 2017;8:15008.

15. Cartharius K, et al. MatInspector and beyond: promoter analysis based on transcription factor binding sites. *Bioinformatics* 2005;21(13):2933–2942.

16. Hu Y, Smyth GK. ELDA: extreme limiting dilution analysis for comparing depleted and enriched populations in stem cell and other assays. *J. Immunol. Methods* 2009;347(1–2):70–78.

17. Di Cello F, et al. Cyclooxygenase inhibitors block uterine tumorigenesis in HMGA1a transgenic mice and human xenografts. *Mol. Cancer Ther.* 2008;7(7):2090–2095.

18. Belton A, et al. HMGA1 induces intestinal polyposis in transgenic mice and drives tumor progression and stem cell properties in colon cancer cells. *PLoS One* 2012;7(1):e30034.

19. Hillion J, et al. The High Mobility Group A1 (HMGA1) gene is highly overexpressed in human uterine

serous carcinomas and carcinosarcomas and drives Matrix Metalloproteinase-2 (MMP-2) in a subset of tumors. *Gynecol. Oncol.* 2016;141(3):580–587.

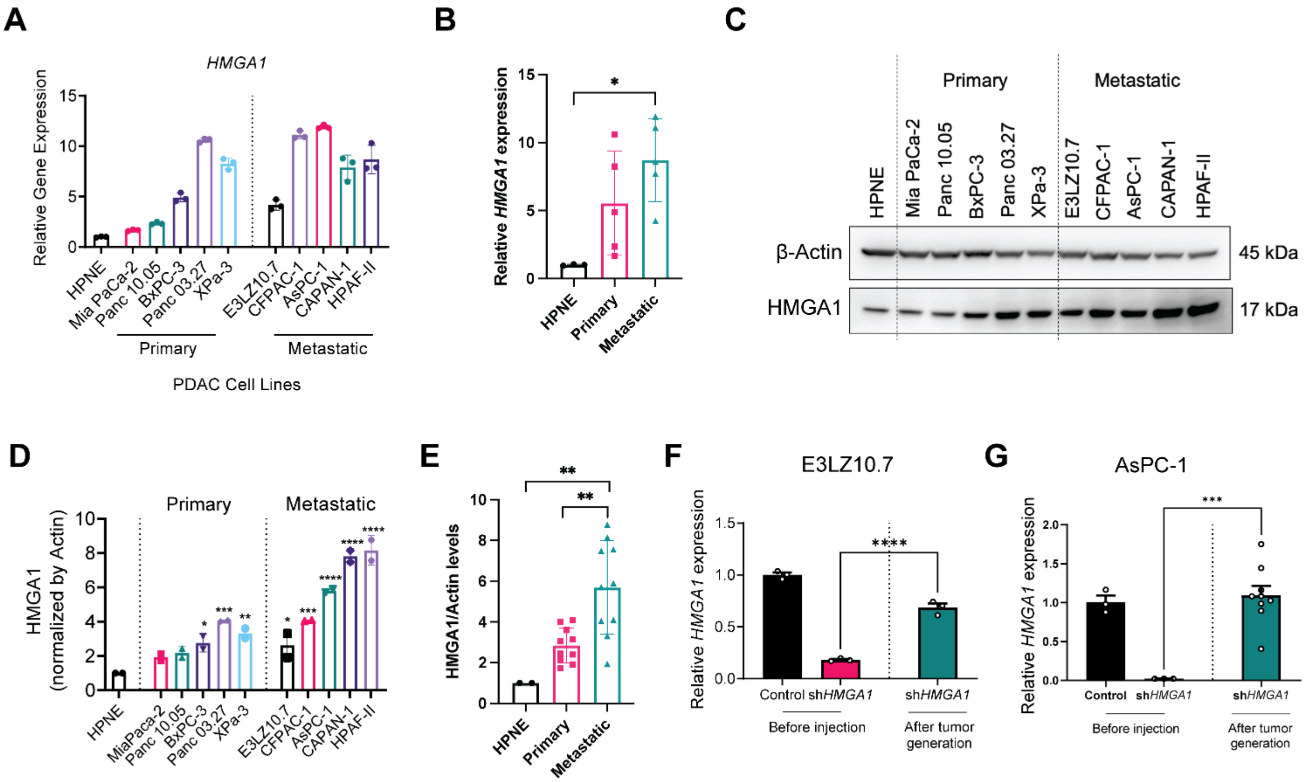

H

Number of Cells Transplanted	E3LZ10.7 + Control	E3LZ10.7 + sh*HMGA1*
1.0 x 10^5	8/10	8/10
5.0 x 10^4	7/10	3/10
1.0 x 10^4	8/10	0/10
**Stem Cell Frequency**	1 in 33, 271	1 in 95, 444
**95% CI**	1/56450 – 1/19610	1/172765 – 1/52728
**P - Value**	0.00552	

I

Number of Cells Transplanted	AsPC-1 + Control	AsPC-1 + sh*HMGA1*
1.0 x 10^5	10/10	7/10
5.0 x 10^4	10/10	7/10
1.0 x 10^4	9/10	5/10
**Stem Cell Frequency**	1 in 4, 343	1 in 47, 616
**95% CI**	1/9, 729 – 1/1, 939	1/80, 433 – 1/28, 188
**P - Value**	0.000000499	

Supplemental Figure 1. ***HMGA1* is overexpressed in human PDAC cells, and silencing *HMGA1* depletes tumor initiator cells**.
(A) *HMGA1* expression in PDAC cells lines from primary (localized) and metastatic PDAC and non-transformed, *TERT*-immortalized HPNE cells from 1 experiment performed in triplicate.
(B) *HMGA1* expression in primary PDAC, metastatic PDAC, and HPNE cell lines.
(C) Representative immunoblots (n=2 experiments) of HMGA1 in primary PDAC, metastatic PDAC, and HPNE cell lines.
(D) Densitometry analysis of HMGA1 levels normalized to β-Actin of immunoblots in PDAC cell lines (from C).
(E) HMGA1 protein levels in primary PDAC, metastatic PDAC, and HPNE cell lines.
(F) *HMGA1* expression in cells with *HMGA1* silencing before injection and from tumors that formed following injection (n=3 tumors from E3LZ10.7 cells with *HMGA1* silencing).
(G) *HMGA1* expression in cells with *HMGA1* silencing before injection and from tumors that formed following injection (n=7 tumors from AsPC-1 cells with *HMGA1* silencing).
(H) Xenograft tumorigenicity at limiting dilutions in E3LZ10.7 ± *HMGA1* silencing. Tumor initiator cell frequency assessed by extreme limiting dilution analysis (ELDA; n=10/condition). Data show 95% confidence interval around tumor initiator cell frequency. *P* values determined by chi-square test.
(I) Xenograft tumorigenicity at limiting dilutions in AsPC-1 ± *HMGA1* silencing. Tumor initiator cell frequency assessed by ELDA (n=10/condition). Data show 95% confidence interval around tumor initiator cell frequency. *P* values determined by chi-square test.
Data shown as mean ± SD. *P* values determined by one-way ANOVA with Dunnett's multiple comparisons **(A, B, D, E)** or Turkey's multiple comparisons **(F, G).** *$P < 0.05$, **$P < 0.01$, ****$P < 0.0001$.

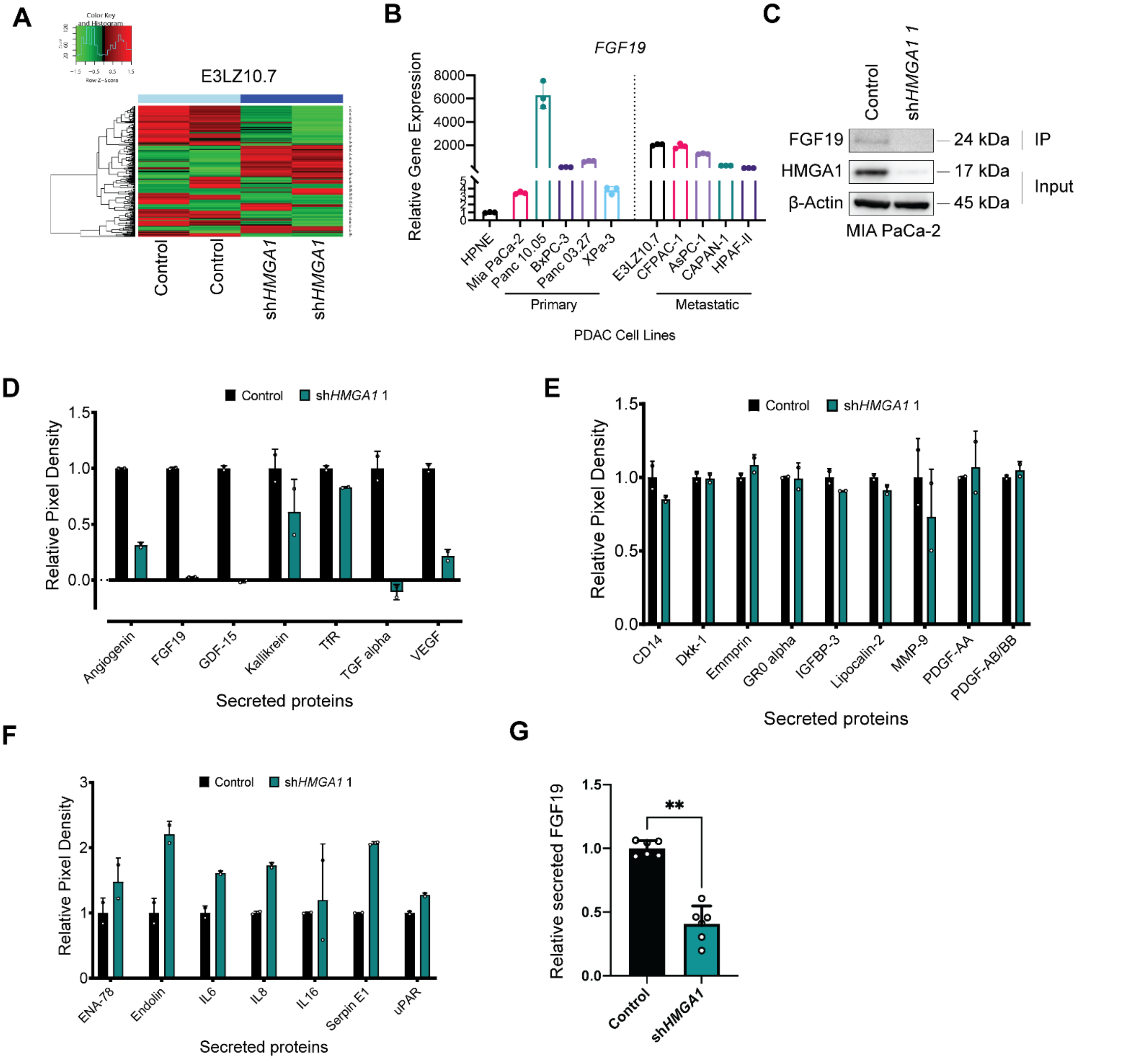

Supplemental Figure 2. **HMGA1 induces the expression and secretion of FGF19.**
(A) Unsupervised clustering analysis of differentially expressed genes (DEGs) by RNA sequencing in E3LZ10.7 cells ± *HMGA1* silencing (performed in duplicate).
(B) *FGF19* expression in primary PDAC, metastatic PDAC, and HPNE cell lines performed once in triplicate.
(C) Representative immunoblot (n=3 experiments) of FGF19 after immunoprecipitation (IP) in MIA PaCa-2 cells ± *HMGA1* silencing.
(D) Secreted cytokine profile of factors repressed with *HMGA1* silencing in E3LZ10.7 cells from a single cytokine array per condition (control versus *HMGA1* silencing via sh*HMGA1* 1 or sh*HMGA1* 2).
(E) Secreted cytokine profile of factors that are unchanged with *HMGA1* silencing in E3LZ10.7 cells from a single cytokine array per condition (control versus *HMGA1* silencing via sh*HMGA1* 1 or sh*HMGA1* 2).
(F) Secreted cytokine profile of factors that are induced with *HMGA1* silencing in E3LZ10.7 cells from a single cytokine array per condition (control versus *HMGA1* silencing via sh*HMGA1* 1 or sh*HMGA1* 2).
(G) ELISA results showing secreted FGF19 in MIA PaCa-2 cells ± *HMGA1* silencing.
Data shown as mean ± SD. *P* values determined by Mann-Whitney test. $^{**}P < 0.01$ (n=3 experiments performed in duplicate).

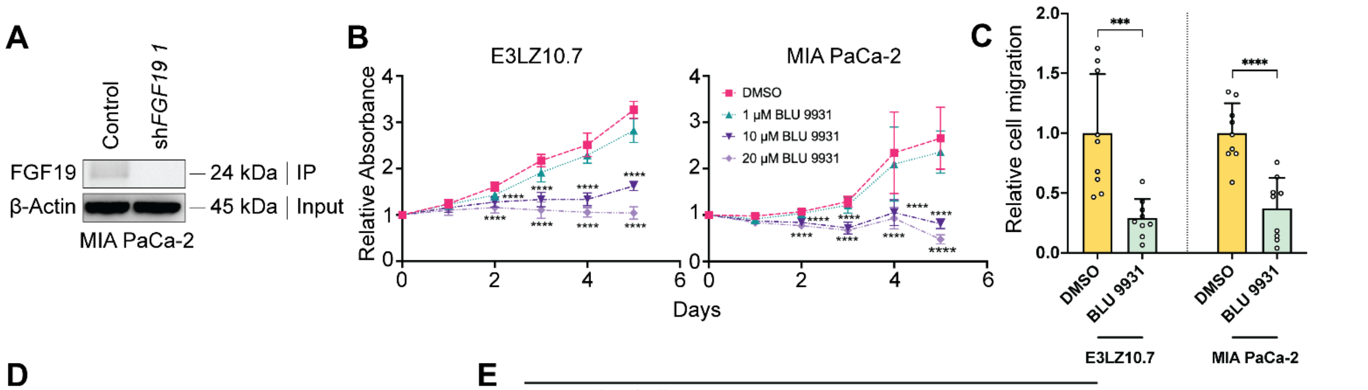

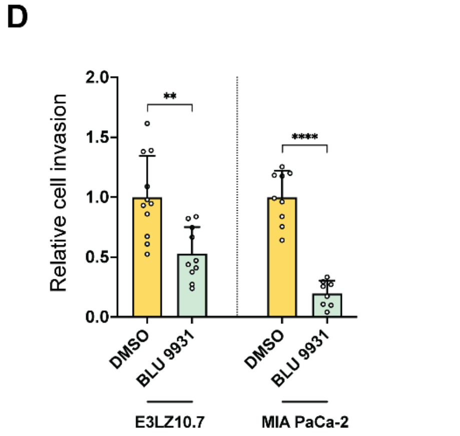

E

Number of Cells Transplanted	E3LZ10.7 + Control	E3LZ10.7 + sh*FGF19*
1.0×10^{5}	8/10	8/10
5.0×10^{4}	7/10	4/10
1.0×10^{4}	8/10	1/10
**Stem Cell Frequency**	1 in 33, 271	1 in 76, 908
**95% CI**	1/56450 – 1/19610	1/134904 – 1/43845
**P - Value**	0.0229	

F

Number of Cells Transplanted	AsPC-1 + Control	AsPC-1 + sh*FGF19*
1.0×10^{5}	10/10	9/10
5.0×10^{4}	10/10	8/10
1.0×10^{4}	9/10	6/10
**Stem Cell Frequency**	1 in 4, 343	1 in 27, 586
**95% CI**	1/9, 729 – 1/1, 939	1/47, 494 – 1/16, 023
**P - Value**	0.00016	

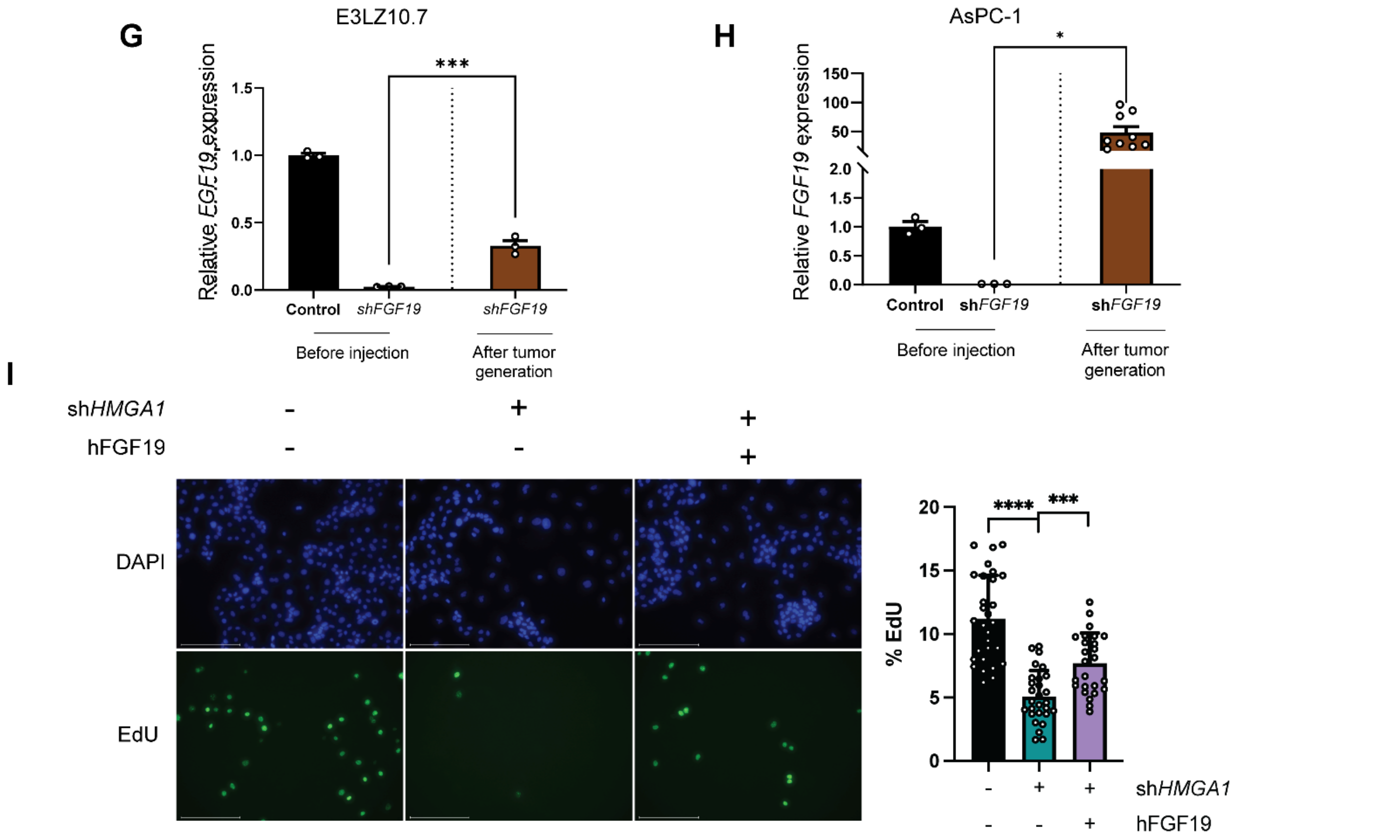

Supplemental Figure 3. FGF19 is required for proliferation and tumorigenesis in PDAC cells.
(A) Representative immunoblot (n=3 experiments) of FGF19 after IP in MIA PaCa-2 cells ± *FGF19* silencing.
(B) MTT proliferation assays comparing PDAC cells ± BLU 9931 treatment from 2 experiments performed in triplicate.

(C) Transwell migration assay comparing PDAC cells ± BLU 9931 treatment (Mia PaCa-2: 10 μM; E3LZ10.7: 20 μM) from 3 experiments performed in triplicate.

(D) Transwell invasion assay comparing PDAC cells ± BLU 9931 treatment (Mia PaCa-2: 10 μM; E3LZ10.7: 20 μM) from 3 experiments performed in triplicate.

(E) Xenograft tumorigenicity at limiting dilutions in E3LZ10.7 ± *FGF19* silencing. Tumor initiator frequency assessed by ELDA (n=10/condition). Data show 95% confidence interval around tumor cell initiator frequency. *P* values determined by chi-square test.

(F) Xenograft tumorigenicity at limiting dilutions in AsPC-1 ± *FGF19* silencing. Tumor initiator cell frequency assessed by ELDA (n=10/condition). Data show 95% confidence interval around tumor initiator cell frequency. *P* values determined by chi-square test.

(G) *FGF19* expression in cells with *FGF19* silencing before injection and from tumors that formed following injection (n=3 tumors from E3LZ10.7 cells with *FGF19* silencing).

(H) *FGF19* expression in cells with *FGF19* silencing before injection and from tumors that formed following injection (n=9 tumors from AsPC-1 cells with *FGF19* silencing).

(I) EdU (5-ethynyl-2'-deoxyuridine) incorporation assay comparing proliferation of *HMGA1* silenced E3LZ10.7 cells ± human FGF19 protein (hFGF19, 100 ng/ml). Data from 5 fields per sample from 2 experiments performed in triplicate.

Data from in vitro assays show mean ± SD. *P* values determined by student's t-test **(C, D),** one-way ANOVA with Turkey's multiple comparisons **(G, H),** or Dunnett's multiple comparisons **(B, I)**. $*P < 0.05$, $**P < 0.01$, $***P < 0.001$, $****P < 0.0001$. Scale bar: 200 μm.

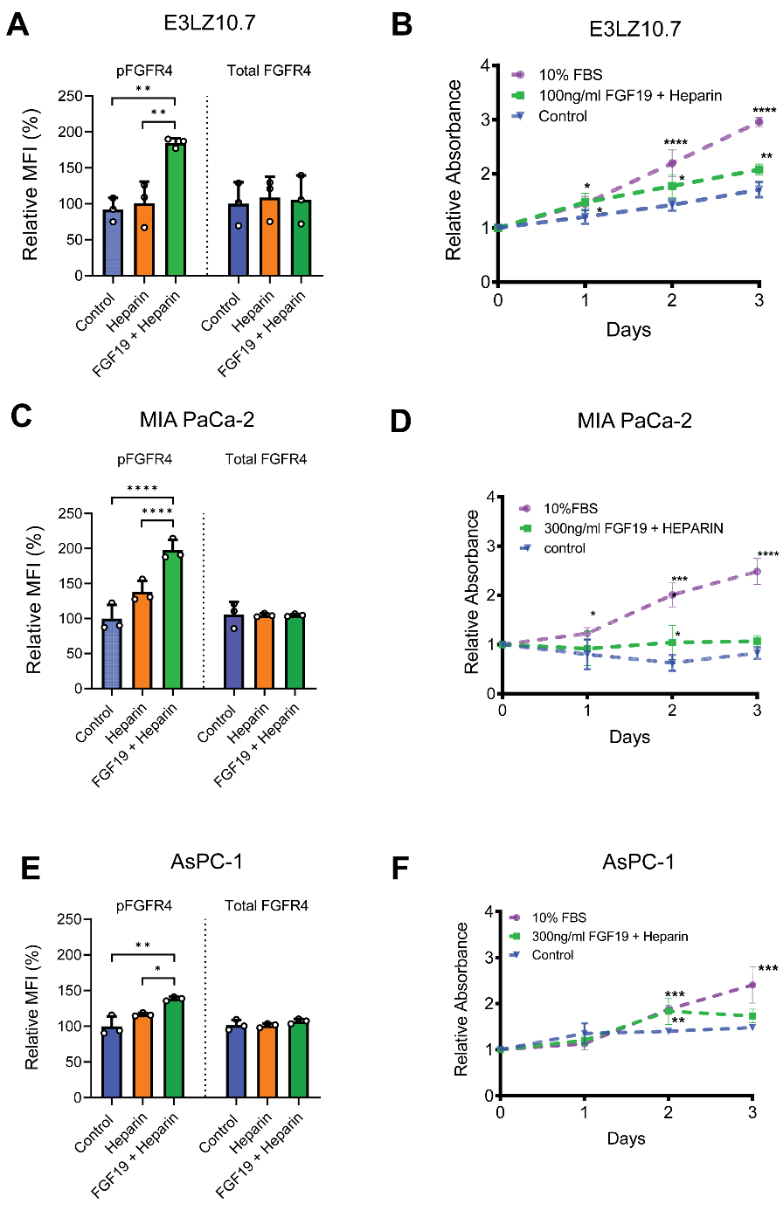

Supplemental Figure 4. Recombinant FGF19 induces phosphorylation of FGFR4 and proliferation in PDAC cell lines.

(A) Relative MFI of pFGFR4 and total FGFR4 in E3LZ10.7 cells from 1 experiment performed with 3 biological replicates.

(B) MTT proliferation assays comparing E3LZ10.7 cells ± recombinant FGF19 (100 ng/ml) from 1 experiment performed in triplicate.

(C) Relative MFI of pFGFR4 and total FGFR4 in MIA PaCa-2 cells from 1 experiment performed with 3 biological replicates.

(D) MTT proliferation assays comparing MIA PaCa-2 cells ± recombinant FGF19 (300 ng/ml required for phosphorylation of FGFR4 in MIA PaCa-2 cells) from 1 experiment performed in triplicate.

(E) Relative MFI of pFGFR4 and total FGFR4 in AsPC-1 cells from 1 experiment performed with 3 biological replicates.

(F) MTT proliferation assays comparing AsPC-1 cells ± recombinant FGF19 (300 ng/ml required for phosphorylation of FGFR4 in AsPC-1) from 1 experiment performed in triplicate.

Data shown as mean ± SD. *P* values determined by one-way ANOVA with Dunnett's multiple comparisons **(A-F)**. **P* < 0.05, ***P* < 0.01, ****P* < 0.001, *****P* < 0.0001.

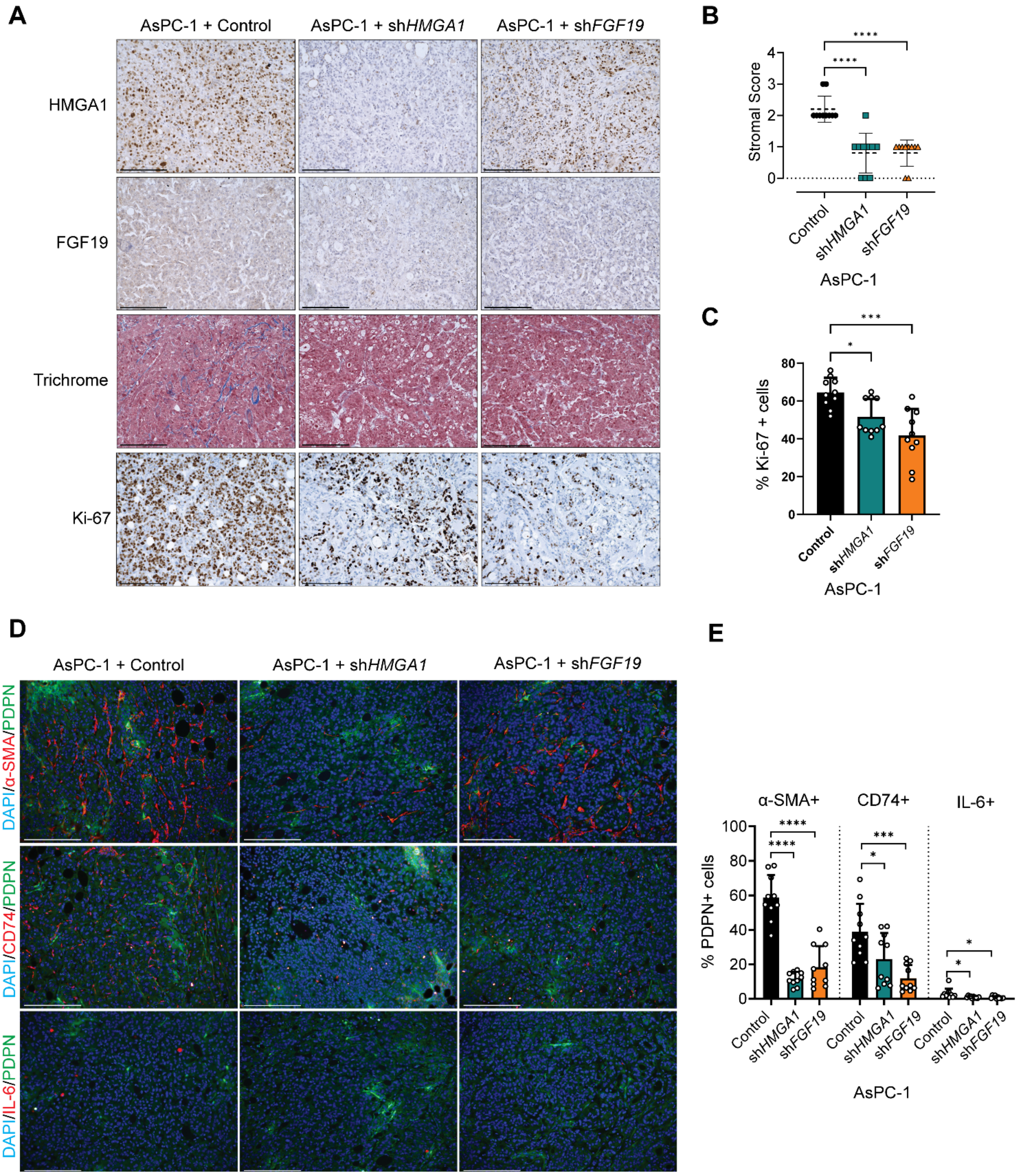

Supplemental Figure 5. HMGA1 and FGF19 induce fibrotic stroma formation, proliferation (Ki-67), and modulate CAF composition during PDAC xenograft tumorigenesis (AsPC-1 cells) tumorigenesis.

(A) Representative images (n=10 per condition) of HMGA1 (IHC, top row), FGF19 (IHC, second row), fibrosis (trichrome, third row), and Ki-67 (IHC, bottom row) in AsPC-1 xenografts ± *HMGA1* or ± *FGF19* silencing.

(B) Quantitative comparison of stroma and CAF composition in AsPC-1 xenografts ± *HMGA1* or *FGF19* silencing. Fibrosis score based on a 3-point system (<5% = 0, 5-30% = 1, 30-60% = 2, >60% = 3). Data from 5 fields at 20X magnification from tumors with 2 mice/group; n=10 per condition.

(C) Comparison of Ki-67 positive cell number in xenografts from 5 fields at 20X magnification from tumors with 2 different mice/group, n=10 per condition.

(D) Representative IF images and **(E)** comparison of CAF composition in AsPC-1 xenografts ± *HMGA1* or ± *FGF19* silencing. Total CAF number ascertained by co-stain with DAPI and PDPN; α-SMA, CD74, or IL6 stains

used to identity percentage of total CAFs positive for each marker from 5 fields at 20X magnification in tumor sections from 2 different mice/group, n=10 per condition.
Data presented as mean ± SD. *P* values determined by ANOVA with Dunnett's multiple comparisons **(B, C, E)**. $^{*}P < 0.05$, $^{**}P < 0.01$, $^{***}P < 0.001$, $^{****}P < 0.0001$. Scale bar: 200 μm.

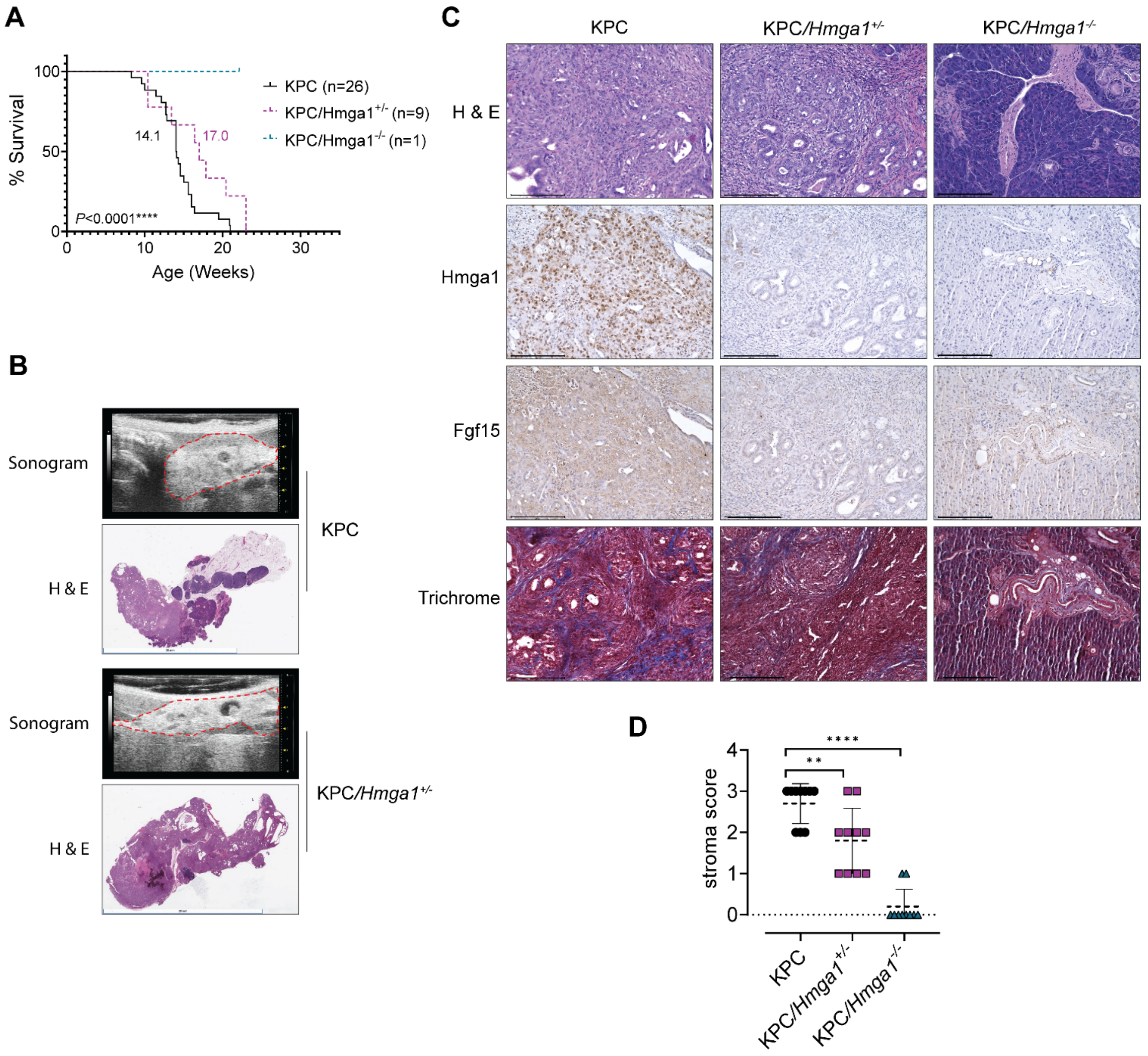

Supplemental Figure 6. Hmga1 and Fgf15 associate with fibrotic stromal formation.

(A) Kaplan-Meier plot showing survival of KPC mice (n=26, 11 males) compared to KPC with global heterozygous *Hmga1* deficiency, KPC/*Hmga*$^{+/-}$ (n=9, 3 males), or KPC mice with global homozygous *Hmga1* deficiency KPC/*Hmga1*$^{-/-}$ (n=1, male). Median survivals are indicated. *P* values determined by Log-rank (Mantel-Cox) test.

(B) Ultrasound (top) and H&E (bottom) of pancreas from KPC mice with or without global *Hmga1* deficiency. KPC mouse was 16 weeks (female); KPC/*Hmga1*$^{+/-}$ mouse was 20.4 weeks (male).

(C) Representative images (n=10 per condition) of H&E (top row), Hmga1 (second column), Fgf15 (third column), and fibrosis (trichrome; bottom row) in PDAC lesions of KPC mouse at 16 weeks (female), KPC/*Hmga1*$^{+/-}$ mouse at 16.4 weeks (female), and pancreas from KPC/*Hmga*$^{-/-}$ mouse at 22.1 weeks (male). Scale bar: 200 μm.

(D) Fibrosis scores in KPC models based on a 3-point system (<5% = 0, 5-30% = 1, 30-60% = 2, >60% = 3) from 10 fields at 20X magnification (n=10 per condition).

P values determined by one-way ANOVA with Dunnett's multiple comparisons **(D)**. **P* < 0.05, ***P* < 0.01, ****P* < 0.001, *****P* < 0.0001. Scale bar: 200 μm.

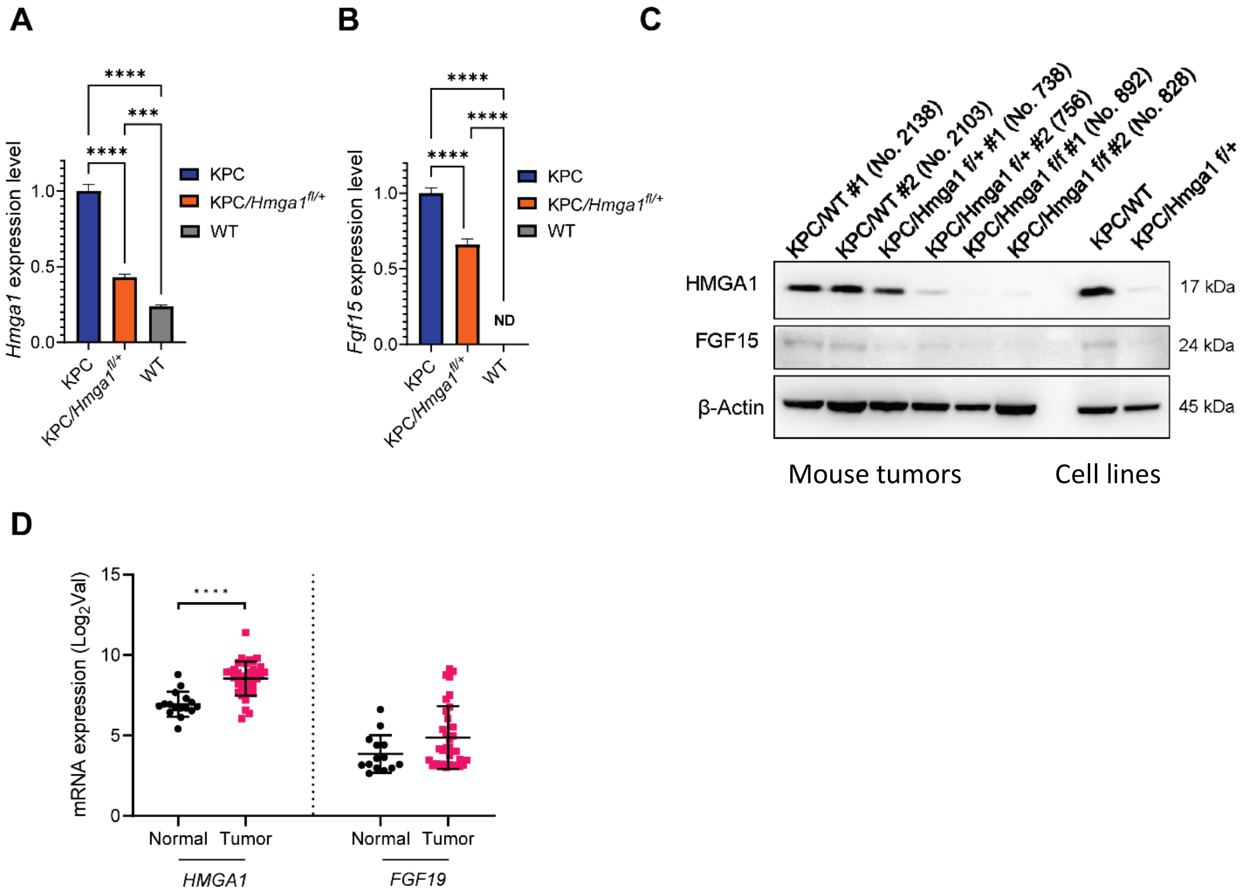

Supplemental Figure 7. *HMGA1/Hmga1* and *FGF19/Fgf15* expression in KPC mice and human PDAC.
(A) *Hmga1* expression in cell lines derived from pancreatic tumors of KPC or KPC/*Hmga1*$^{fl/+}$ mice and wild type (WT) snap-frozen pancreatic tissue. Experiment performed twice in triplicate.
(B) *Fgf15* expression in cell lines derived from pancreatic tumors of KPC or KPC/*Hmga1*$^{fl/+}$ mice and wild type (WT) snap-frozen pancreatic tissue. Experiment was performed twice in triplicate.
(C) Representative immunoblots (n=2 experiments) of Hmga1 and Fgf15 protein levels in mouse pancreatic tumors of KPC, KPC/*Hmga1*$^{fl/+}$, and KPC/*Hmga1*$^{fl/fl}$ mice (left 6 lanes) or from cell lines derived from KPC and KPC/*Hmga1*$^{fl/+}$ mice (right 2 lanes).
(D) *HMGA1* and *FGF19* mRNA levels in normal pancreatic tissue and primary PDAC (GSE16515); n=36 for PDAC and n=16 for normal control pancreas.
Data shown as mean ± SD. ***P*** values determined by one-way ANOVA with Turkey's multiple comparisons **(A-B)** and student t-test **(D).** ***P < 0.001, ****P < 0.0001

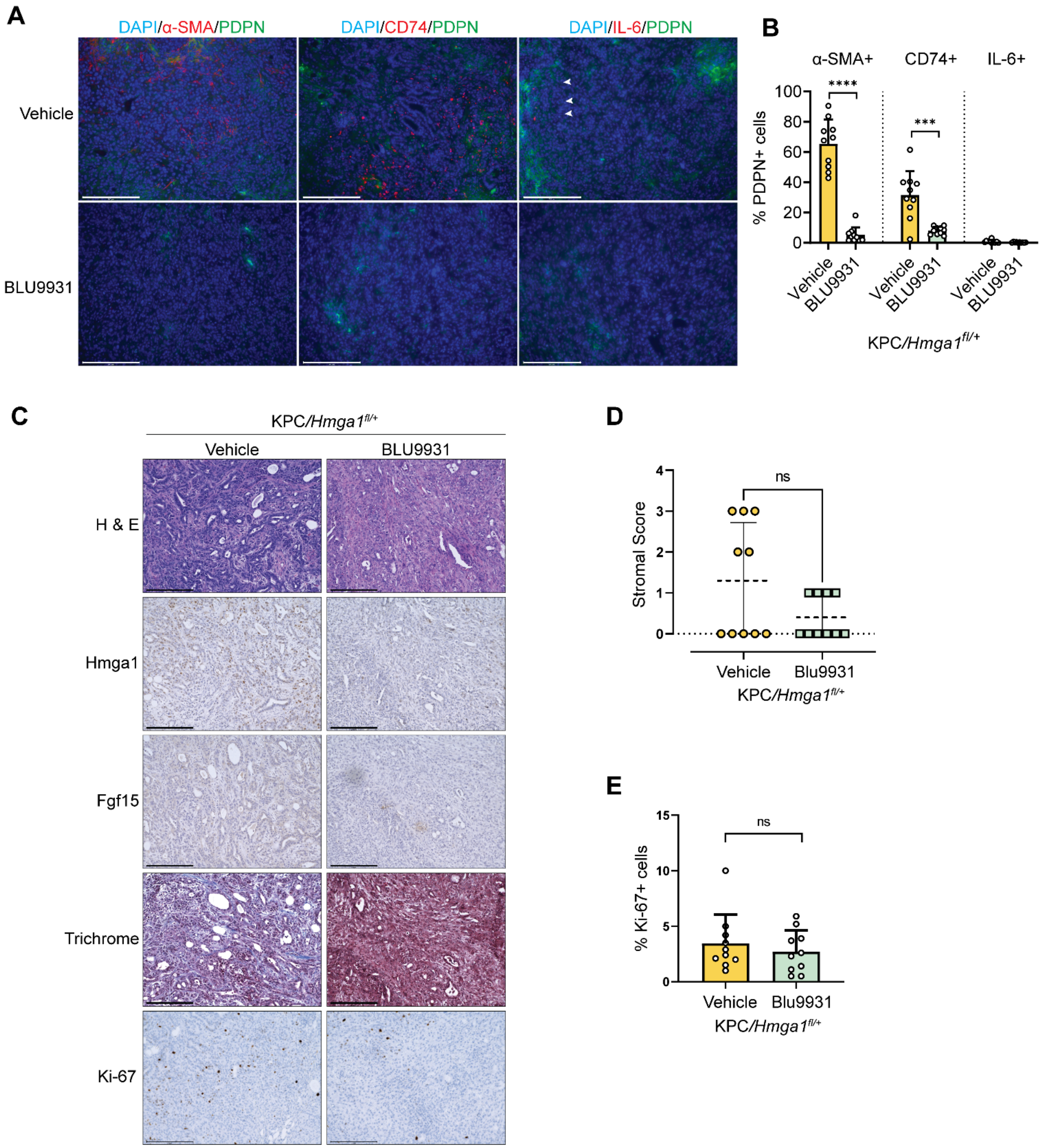

Supplemental Figure 8. BLU9931 effects on tumorigenesis and stroma formation in orthotopic implants from KPC cells with pancreas-specific *Hmga1* heterozygous deficiency.

(A) Representative IF (n=10/condition) comparing CAFs from KPC/*Hmga1*$^{+/-}$ orthotopic implants of mice treated with BLU9931 or vehicle control.

(B) CAF composition comparisons in KPC/*Hmga1*$^{+/-}$ orthotopic implants of mice treated with BLU9931 or vehicle control. Total CAF number ascertained by co-stain with DAPI and PDPN. CAF composition calculated from PDPN CAFs that also stain for one of the following: α-SMA, CD74, or IL6 from 10 fields at 20X magnification (n=10 per condition). *P* values determine by student's t-test for CD74+ CAF (data normally distributed) and Mann-Whitney for α-SMA+ and IL6+ CAF (data not normally distributed).

(C) Representative images (n=10 per condition) of tumors by H&E (top row), Hmga1 (second row), Fgf15 (third row), fibrosis (trichrome, fourth row), and Ki-67 (bottom row) in KPC/*Hmga1*$^{+/-}$ orthotopic implants of mice treated with BLU9931 or vehicle control.

(D) Stromal fibrosis scores in KPC/*Hmga1*$^{+/-}$ orthotopic implants based on a 3-point system (<5% = 0, 5-30% = 1, 30-60% = 2, >60% = 3) from 10 fields at 20X magnification of tumors from 3 different mice/group (n=10 per condition).
(E) Comparison of Ki-67 positive cells in KPC orthotopic implants of mice treated with BLU9931 or vehicle control from a total of 10 fields at 20X magnification of tumors from 3 different mice/group (n=10 per condition).
Data shown as mean ± SD. *P* values determined by Mann-Whitney Test **(D, E)**. $^{*}P < 0.05$, $^{**}P < 0.01$, $^{***}P < 0.001$, $^{****}P < 0.0001$. Scale bar: 200 μm.

Supplemental Table 1. GSEA of MSigDb Hallmark and curated pathways associated with HMGA1 in E3LZ10.7 cells.

Supplemental Table 2. Medium for cell culture and cell line establishment

Medium for cancer and CAF cell lines

Item	Company/Cat	Final Conc	Cell lines
DMEM	Gibco/ 11965118	-	E3LZ10.7, MIA Paca-2
FBS	Corning/ 35-011-CV	10%	
RPMI-1640	Gibco/ 11875119	-	AsPC-1
FBS	Corning/ 35-011-CV	10%	

Medium for cell line establishment

Item	Company/Cat	Final Conc	Medium type
RPMI-1640	Gibco/ 11875119	-	Transport Medium
Antibiotic-Antimycotic	Gibco/15240062	1X	
Gentamicin	Quality Bio/120-099-661EA	10 μg/mL	
RPMI-1640	Gibco/ 11875119	-	Digestion Medium
FBS	Corning/ 35-011-CV	5%	
Collagenase/Hyaluronidase	StemCell Technologies/07912	1x	
RPMI-1640	Gibco/ 11875119	-	Growth Medium
FBS	Corning/ 35-011-CV	10%	
GlutaMAX	Gibco/35030061	1x	
Non-essential amino acids	MilliporeSigma/M7145-100ML	1x	
Sodium Pyruvate	Gibco/11360070	1x	
Antibiotic-Antimycotic	Gibco/15240062	1X	

Supplemental Table 3. Primers used for gene expression and ChIP studies

Primers for gene expression studies

Primer		Sequence (5' – 3')
Human *HMGA1*	Forward	AGGAAAAGGACGGCACTGAGAA
	Reverse	CCCCGAGGTCTCTTAGGTGTTGG
Human *FGF19*	Forward	CGGAGGAAGACTGTGCTTTCG
	Reverse	CTCGGATCGGTACACATTGTAG
HuPO	Forward	CCATTCTATGATCAACGGGTACAA
	Reverse	AGCAAGTGGGAAGGTGTAATCC
Mouse *Hmga1*	Forward	GCTGGTCGGGAGTCAGAAAG
	Reverse	GGTGACTTTCCGGGTCTTGG
Mouse *Fgf15*	Forward	ATGGCGAGAAAGTGGAACGG
	Reverse	CTGACACAGACTGGGATTGCT
Mouse Gapdh	Forward	AGAAGACTGTGGATGGCCCCTC
	Reverse	GATGACTTGCCCACAGCCTT

Primers for ChIP studies

Primer		Sequence (5' – 3')
h*GAPDH* promoter	Forward	CATCTCAGTCGTTCCCAAAGT
	Reverse	TTCCCAGGACTGGACTGT
h*FGF19* R1 promoter	Forward	GATCGCATCAGCCACTCTCT
	Reverse	AAATGCTCTTGAGGGCGATGG
h*FGF19* R2 promoter	Forward	CCCTCAGTCATTAACGCCAGT
	Reverse	AGACAGCGAGAGAAATGCCA

Supplemental Table 4. Antibodies used for Western blot, ChIP, flow cytometry and immunohistochemistry

Antibodies for Western Blot

Primary Antibody	Clone/Cat #	Company	Dilution
Anti-HMGA1	EPR7839/ab129153	Abcam	1:1000
Anti-FGF19	D1N3R/#83348	Cell Signaling Technology	1:500
Anti-FGF19	H-12/#sc-390621	Santa Cruz Biotechnology	1:250
Anti-pERK	D13.14.4E/#4370	Cell Signaling Technology	1:1000
Anti-pAKT	D9E/#4060	Cell Signaling Technology	1:2000
Anti-ERK	137F5/#4695	Cell Signaling Technology	1:1000
Anti-AKT	C67E7/#4691	Cell Signaling Technology	1:1000
Anti-β-Actin	D6A8/#8457	Cell Signaling Technology	1:1000

Secondary Antibody	Clone/Cat #	Company	Dilution
IRDye 800CW Goat anti-Rabbit IgG	926-32211	LI-COR Biosciences	1:10000
IRDye 680LT Goat anti-Mouse IgG	926-68020	LI-COR Biosciences	1:10000

Antibodies for Immunohistochemistry

Primary Antibody	Clone/Cat #	Company	Dilution
Anti-HMGA1	EPR7839/ab129153	Abcam	1:1000
Anti-FGF19	H-12/sc-390621	Santa Cruz Biotechnology	1:50
Anti-FGF19	ab225942	Abcam	1:100
Anti-FGF15	AF6755	R&D	15μg/ml

Secondary Antibody	Clone/Cat #	Company	Dilution
Donkey anti-Sheep IgG (H+L) Secondary Antibody, HRP	A16041	ThermoFisher Scientific	1:2000
Donkey anti-Rabbit IgG (H+L) Cross-Adsorbed Secondary Antibody, HRP	31458	ThermoFisher Scientific	1:2000

Antibodies for Immunofluorescence

Primary Antibody	Clone/Cat #	Company	Dilution
Anti-PDPN	8.1.1/#127404	BioLegend	1:200
α-SMA	1A4/#48939	Cell Signaling Technology	1:200
Anti-IL6	D5W4V/#12912	Cell signaling Technology	1:200
Anti-CD74	In1/#151002	BioLegend	1:200

Secondary Antibody	Clone/Cat #	Company	Dilution
Streptavidin Alexa Fluor 488	S11223	ThermoFisher Scientific	1:2000
Donkey anti-Mouse IgG (H+L) Alexa Fluor 594	A32744	ThermoFisher Scientific	1:200
Donkey anti-Rabbit IgG (H+L) Alexa Fluor 594	A32754	ThermoFisher Scientific	1:200
Donkey anti-Rat IgG (H+L) Alexa Fluor 594	A48271	ThermoFisher Scientific	1:200

Antibodies for ChIP experiments

Antibody	Clone/Cat #	Company	Amount (μg)
Anti-HMGA1	EPR7839/ab129153	Abcam	1.5
Anti-HMGA1a/HMGA1b	ab4078	Abcam	1.5
Anti-H3K4me3	ab8580	Abcam	3
Anti-H3K27Ac	ab4729	Abcam	3
Anti-H3	ab1719	Abcam	6
Rabbit IgG	NI01-100UG	Millipore Sigma	3

UNCUT GELS

Figure 1B

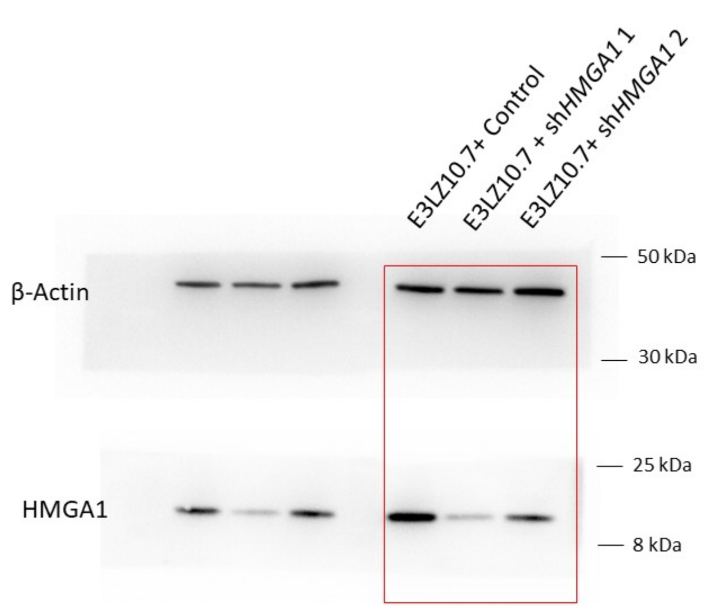

02/17/2021

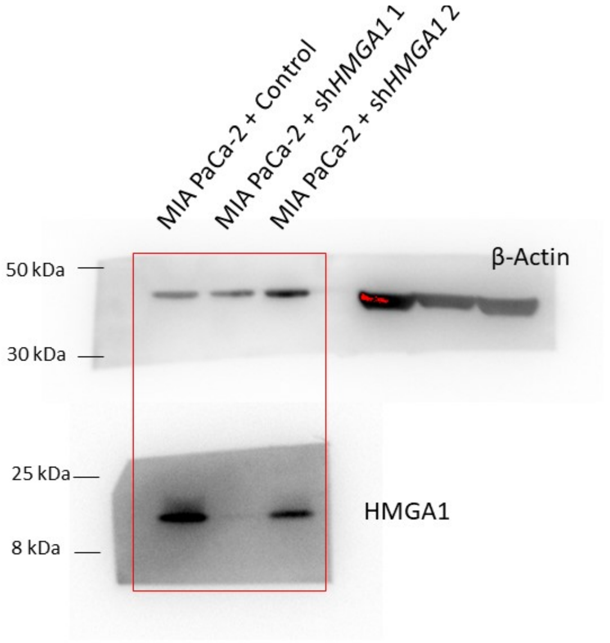

03/03/2021

HMGA1 - EPR7839/ab129153, β-Actin - D6A8/#8457

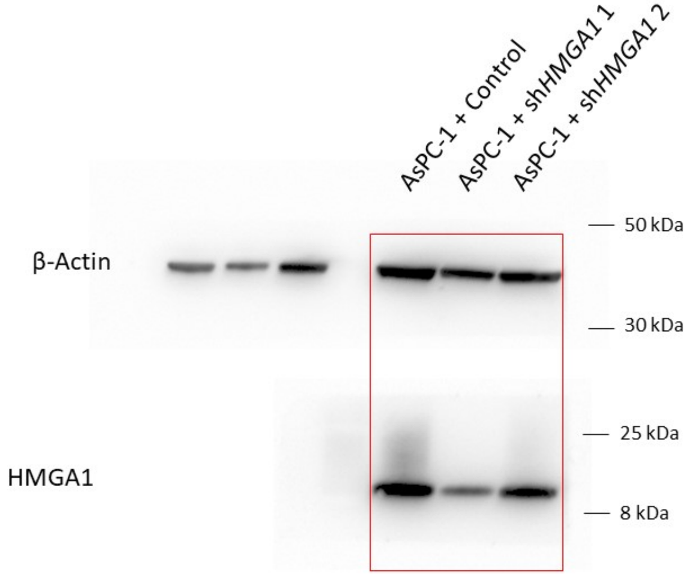

09/21/2021

HMGA1 - EPR7839/ab129153, β-Actin - D6A8/#8457

Figure 3E

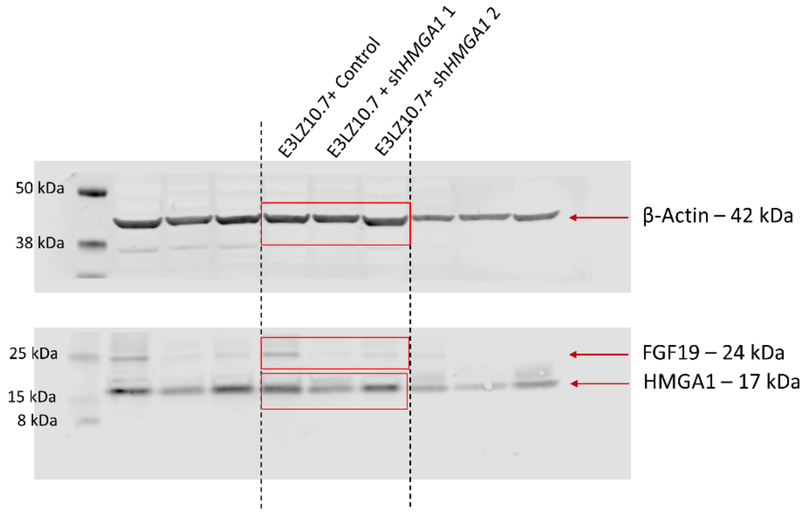

12/18/2020

HMGA1 - EPR7839/ab129153, β-Actin - D6A8/#8457, FGF19 - D1N3R/#83348

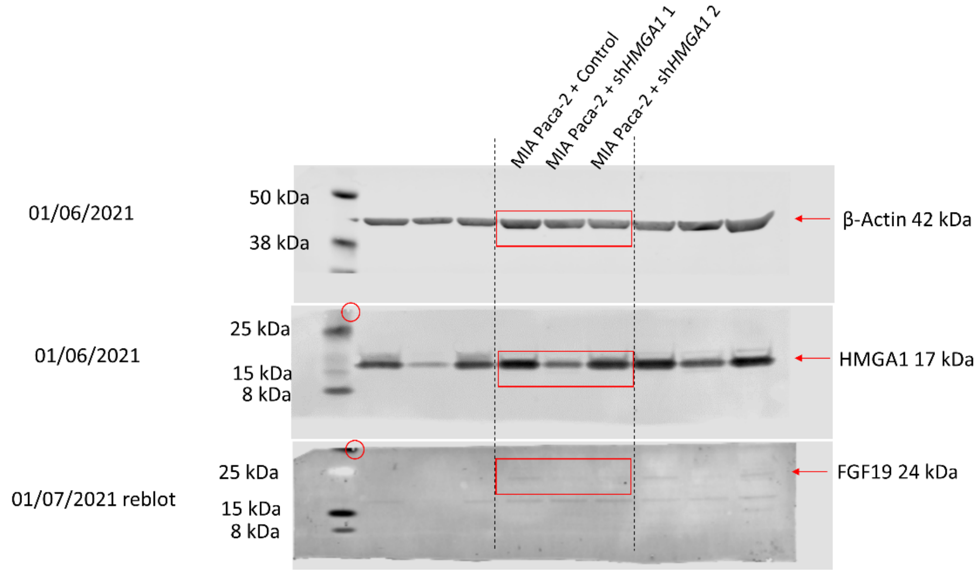

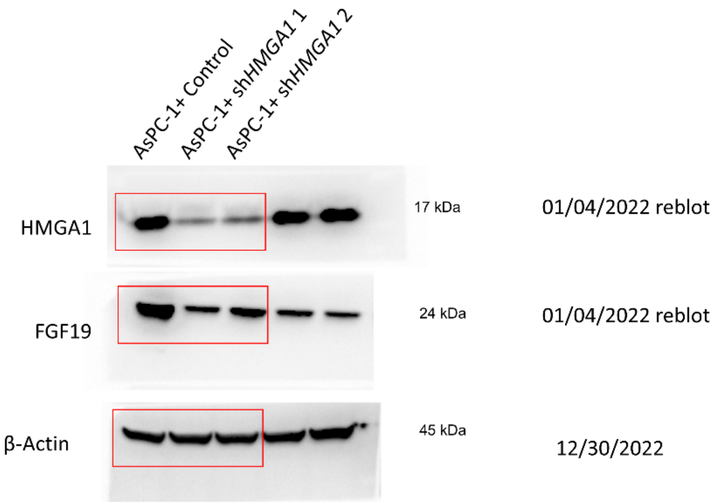

HMGA1 - EPR7839/ab129153,
FGF19 - D1N3R/#83348,
β-Actin - D6A8/#8457

Figure 3H

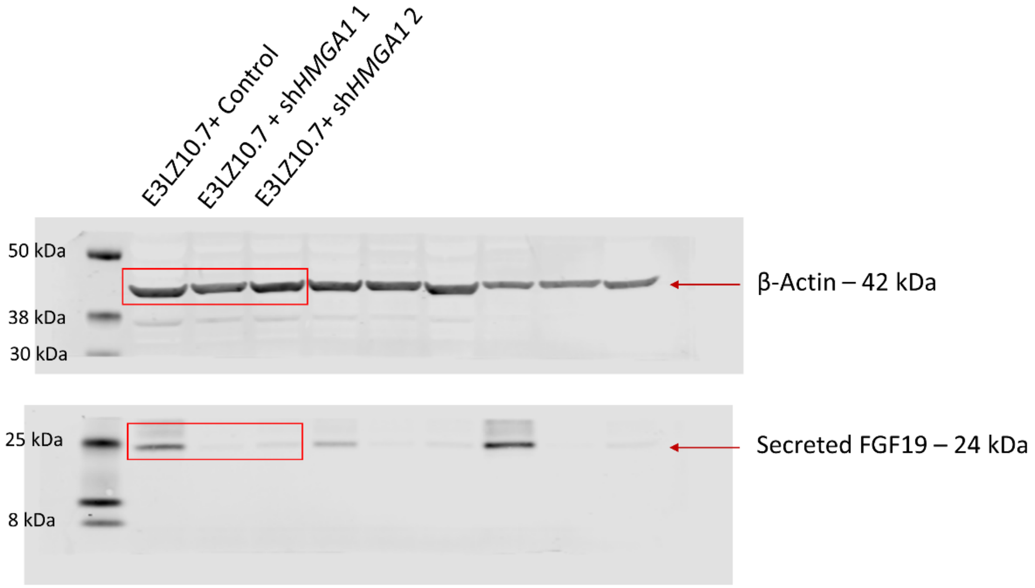

12/23/2020

FGF19 - D1N3R/#83348, β-Actin - D6A8/#8457

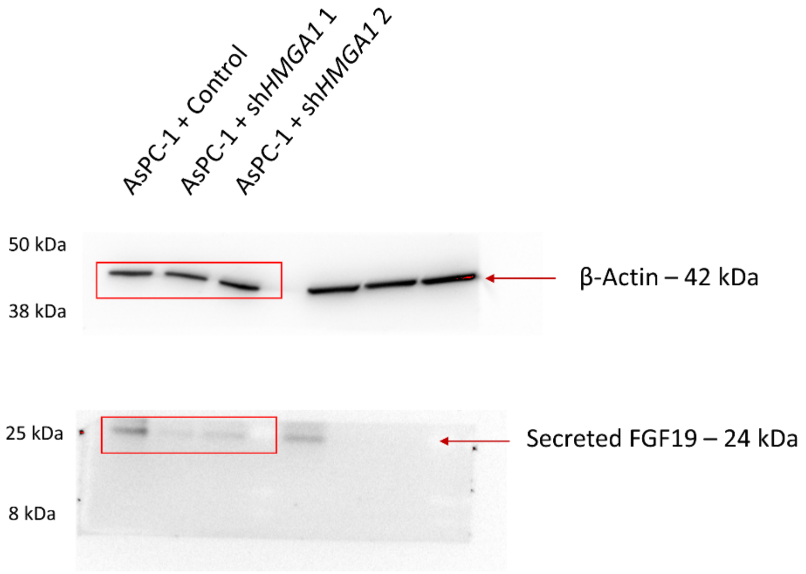

08/21/2021

FGF19 - D1N3R/#83348, β-Actin - D6A8/#8457

Figure 4B

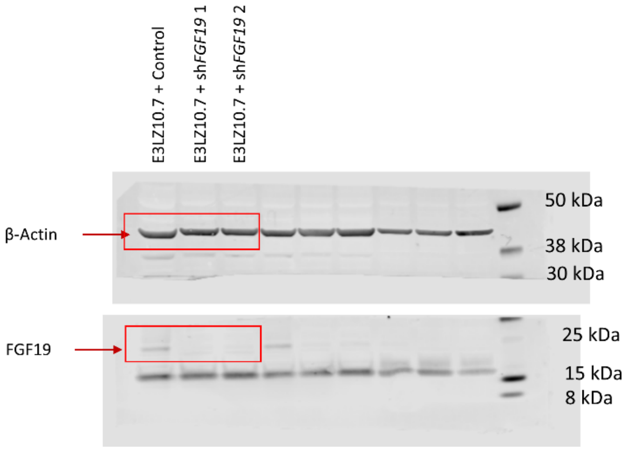

12/18/2020

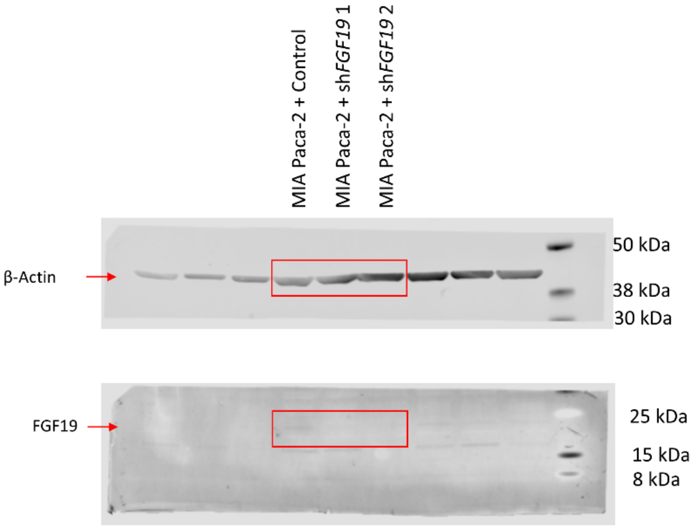

01/06/2021

FGF19 - D1N3R/#83348, β-Actin - D6A8/#8457

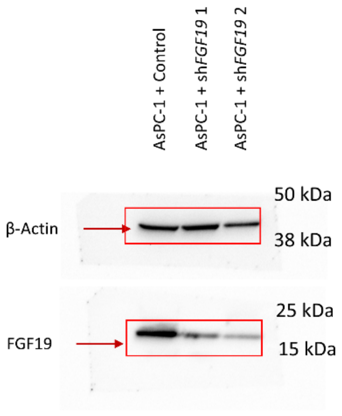

09/01/2021

FGF19 - D1N3R/#83348, β-Actin - D6A8/#8457

Figure 8C

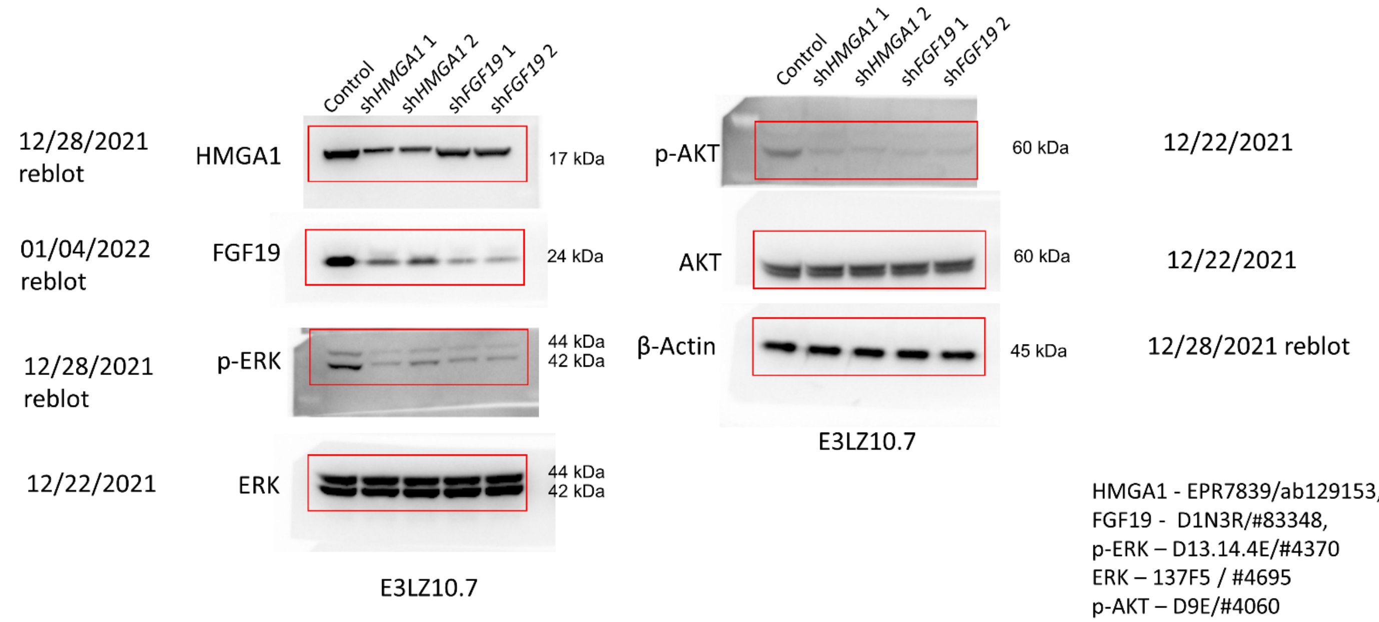

HMGA1 - EPR7839/ab129153,
FGF19 - D1N3R/#83348,
p-ERK – D13.14.4E/#4370
ERK – 137F5 / #4695
p-AKT – D9E/#4060
AKT – C67E7/#4691
β-Actin - D6A8/#8457

Figure 8E

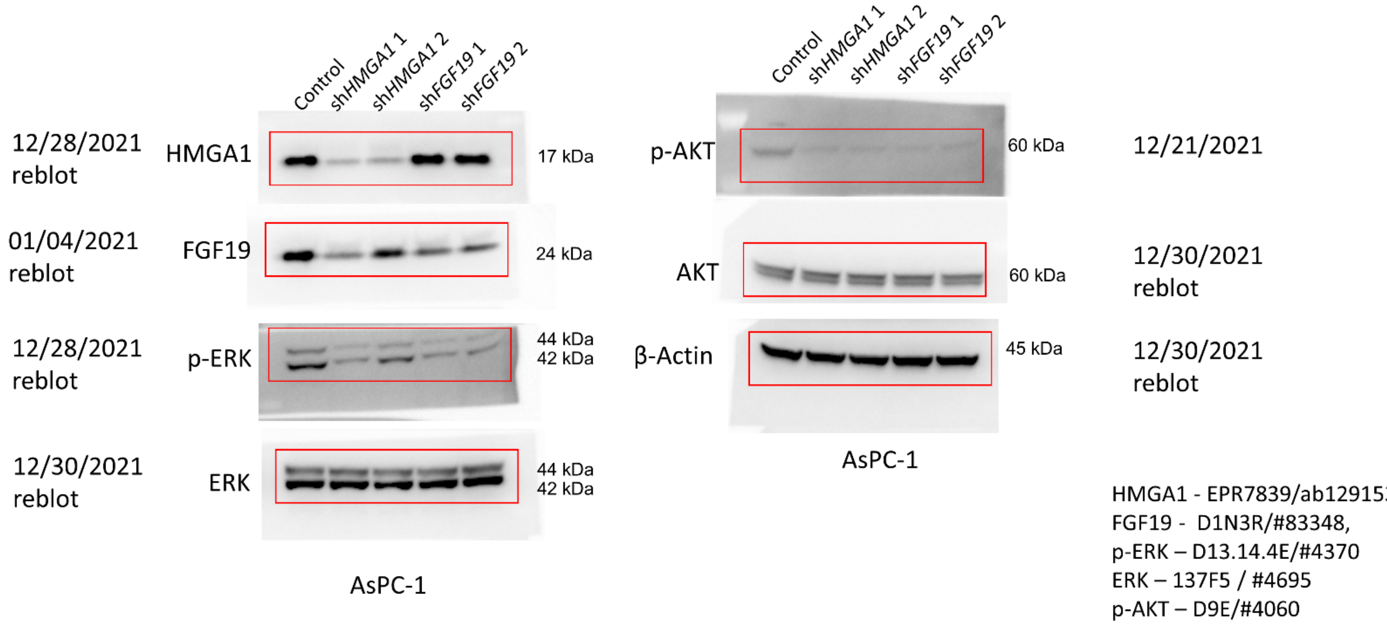

HMGA1 - EPR7839/ab129153,
FGF19 - D1N3R/#83348,
p-ERK – D13.14.4E/#4370
ERK – 137F5 / #4695
p-AKT – D9E/#4060
AKT – C67E7/#4691
β-Actin - D6A8/#8457

Supplemental Figure 1C

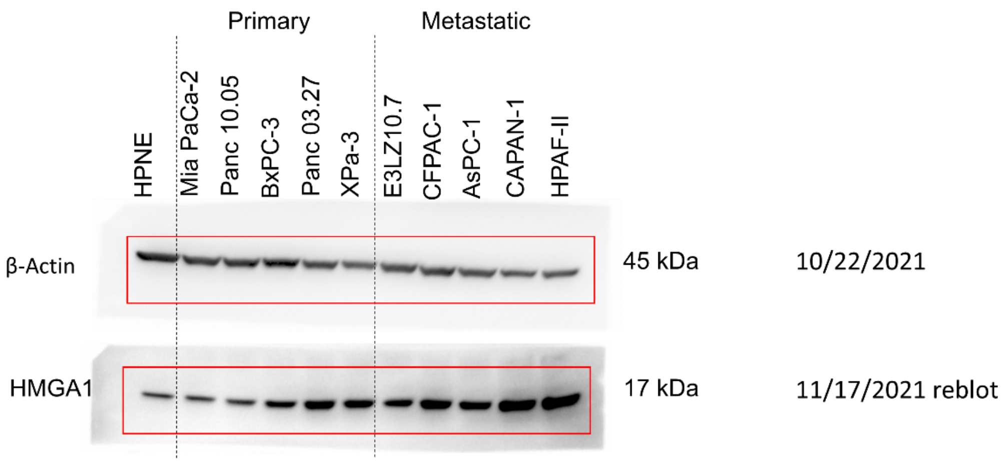

HMGA1 - EPR7839/ab129153, β-Actin - D6A8/#8457

Supplemental Figure 2C

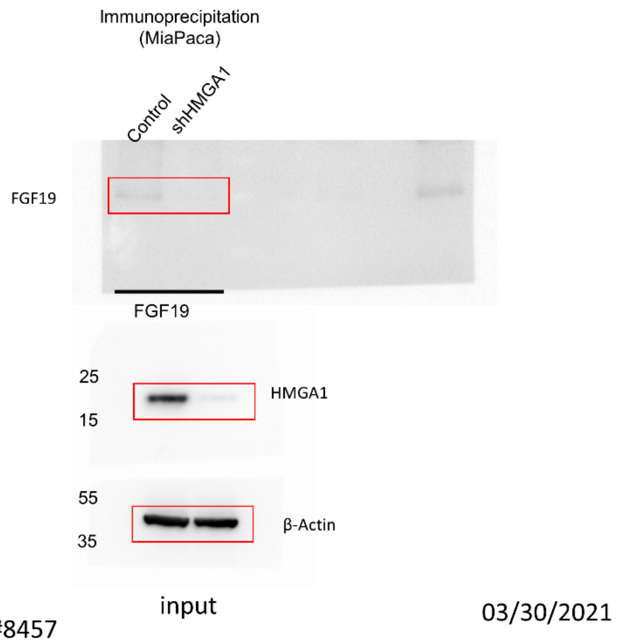

FGF19 - D1N3R/#83348, β-Actin - D6A8/#8457

Supplemental Figure 3A

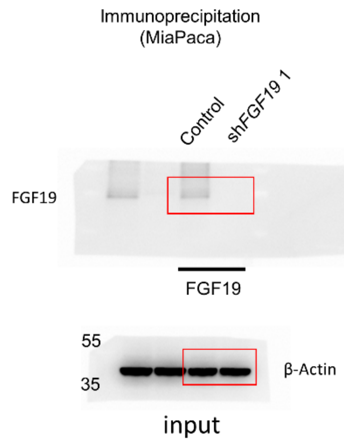

04/13/2021

FGF19 - D1N3R/#83348, β-Actin - D6A8/#8457

Supplemental Figure 7C

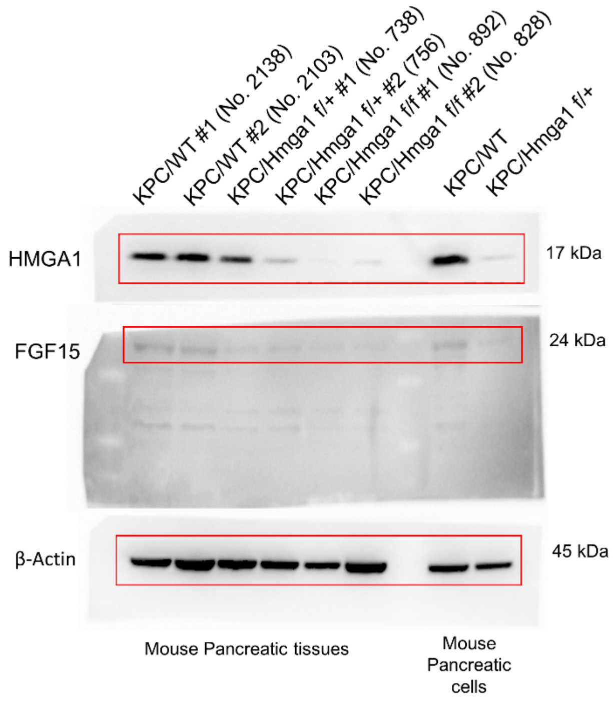

08/02/2022 reblot

08/02/2022 reblot

07/28/2022

HMGA1 - EPR7839/ab129153,
FGF15 - D1N3R/#83348,
β-Actin - D6A8/#8457
